# Extensive hybridization in *Ranunculus* section *Batrachium* (Ranunculaceae) in rivers of two postglacial landscapes of East Europe

**DOI:** 10.1038/s41598-022-16224-0

**Published:** 2022-07-15

**Authors:** Alexander A. Bobrov, Jurgita Butkuvienė, Elena V. Chemeris, Jolanta Patamsytė, Carla Lambertini, Algis Aučina, Zofija Sinkevičienė, Donatas Naugžemys

**Affiliations:** 1 Papanin Institute for Biology of Inland Waters RAS, Borok, Nekouz District, Yaroslavl Region 152742 Russia; 2 Tyumen State University, AquaBioSafe, Lenina Str., 25, Tyumen, 625003 Russia; 3 Botanical Garden, Vilnius University, Kairėnų Str. 43, 01100 Vilnius, Lithuania; 4 Life Sciences Center, Vilnius University, Sauletekio Ave. 7, 10222 Vilnius, Lithuania; 5 Departmanet of Biosciences, Milan University, Via Celoria, 26-Corpo B 20133, Milan, Italy; 6 Institute of Botany, Nature Research Centre, Akademijos Str. 2, 08412 Vilnius, Lithuania

**Keywords:** Plant hybridization, Plant evolution, Genetic hybridization

## Abstract

We demonstrate a wide distribution and abundance of hybrids between the river species *Ranunculus aquatilis*, *R*. *fluitans* and *R*. *kauffmannii* with the still water species *R*. *circinatus* (*Batrachium*, Ranunculaceae) in rivers of two postglacial landscapes of East Europe, i.e., Lithuania and Central European Russia. The *Batrachium* species and hybrid diversity is higher in the rivers of Lithuania (4 species and 3 hybrids vs. 2 and 1) and represented mainly by western *R*. *aquatilis*, *R*. *fluitans* and their hybrids whereas in Central European Russia, the East European species *R*. *kauffmannii* and its hybrid are the only dominant forms. Hybrids make up about 3/4 of the studied individuals found in 3/4 of the studied river localities in Lithuania and 1/3 of the individuals found in 1/3 of the localities in Central European Russia. Such extensive hybridization in river *Batrachium* may have arisen due to the specificity of rivers as open-type ecosystems. It may have been intensified by the transformation of river ecosystems by human activities and the postglacial character of the studied landscapes combined with ongoing climate change. Almost all hybrids of *R*. *aquatilis*, *R*. *fluitans* and *R*. *kauffmannii* originated from unidirectional crossings in which *R*. *circinatus* acted as a pollen donor. Such crossings could be driven by higher frequency and abundance of *R*. *circinatus* populations as well as by some biological mechanisms. Two hybrids, *R*. *circinatus* × *R*. *fluitans* and *R*. *circinatus* × *R*. *kauffmannii*, were formally described as *R.* × *redundans* and *R.* × *absconditus*. We found a hybrid which most likely originated from additional crossing between *R*. *aquatilis* and *R*. *circinatus* × *R*. *fluitans*.

## Introduction

*Ranunculus* L. section *Batrachium* DC. (Ranunculaceae Juss.), hereafter referred to as *Batrachium*, is represented by approximately 30 species occurring in different aquatic and semiaquatic habitats worldwide^1^. The group is regarded as one of the hardest to identify among aquatic plants^2^. In addition to limited diagnostic characters, species delimitation and identification are affected by extreme phenotypic variability, frequent hybridization and polyploidy^1,3–5^.

In *Batrachium,* hybridization significantly increase taxonomic diversity [^6–13^] and is one of the main factors of speciation^1,3–5^.

Cook^6,14^ assumed all members of *Batrachium* to be potentially capable of hybridization, which is supported by recent studies^1,4,5,15^. *Batrachium* hybrids can be partly or fully fertile^1,3–6,14,16^, which can have important evolutionary implications for the reticulate evolution of the group.

Interspecific hybridization plays an important role in formation of plant diversity in general [e.g., ^17–21^]. In many groups of aquatic plants hybridization is one of the main sources of new, stable, and ecologically important taxa [e.g., ^22–29^].

For the identification of *Batrachium*, a large set of morphological characteristics should be considered, but in many cases, additional molecular genetic data complemented by karyological and genome size results can be valuable or even necessary. Despite the wide application of molecular marker polymorphisms for the detection and confirmation of hybrids in several aquatic plant groups, this approach is still uncommon in *Batrachium*^4,5,10–13,29,30^. This is due to both the poorly known diversity of *Batrachium* in many regions as well as the underestimation of the role of hybridization and polyploidization in the evolution of the section.

The highest diversity of hybrids within aquatic plants is found in river ecosystems [e.g., ^17^, ^31–36^], which are characterized by variable environmental conditions and seasonal fluctuations suitable for the colonization by permanently arising new forms. Repeated hybridization events can be more frequent in *Batrachium* in rivers and streams, which play the roles of “evolutionary incubators” for newly arising hybrids and polyploids within this group^4^. Hybrids of aquatic plants have survived in rivers in postglacial areas without parental species since the last glaciation^35^, and human impacts on rivers can induce hybridization^20^.

In addition, *Batrachium* taxa are the main components of river ecosystems protected under the Habitats Directive 92/43/EEC (habitat code 3260)^37^, and *Batrachium* taxa are an indicator of the sustainability of riverine ecosystems^30,38^. This type of habitat is particularly vulnerable to different ecological impacts such as human activity and climate change^38,39^. Therefore, it is important to assess *Batrachium* taxa diversity and population structures to understand levels of genetic diversity and risk of genetic assimilation via hybridization as well as to evaluate the future risk of diversity loss^40,41^.

**Figure 1:**
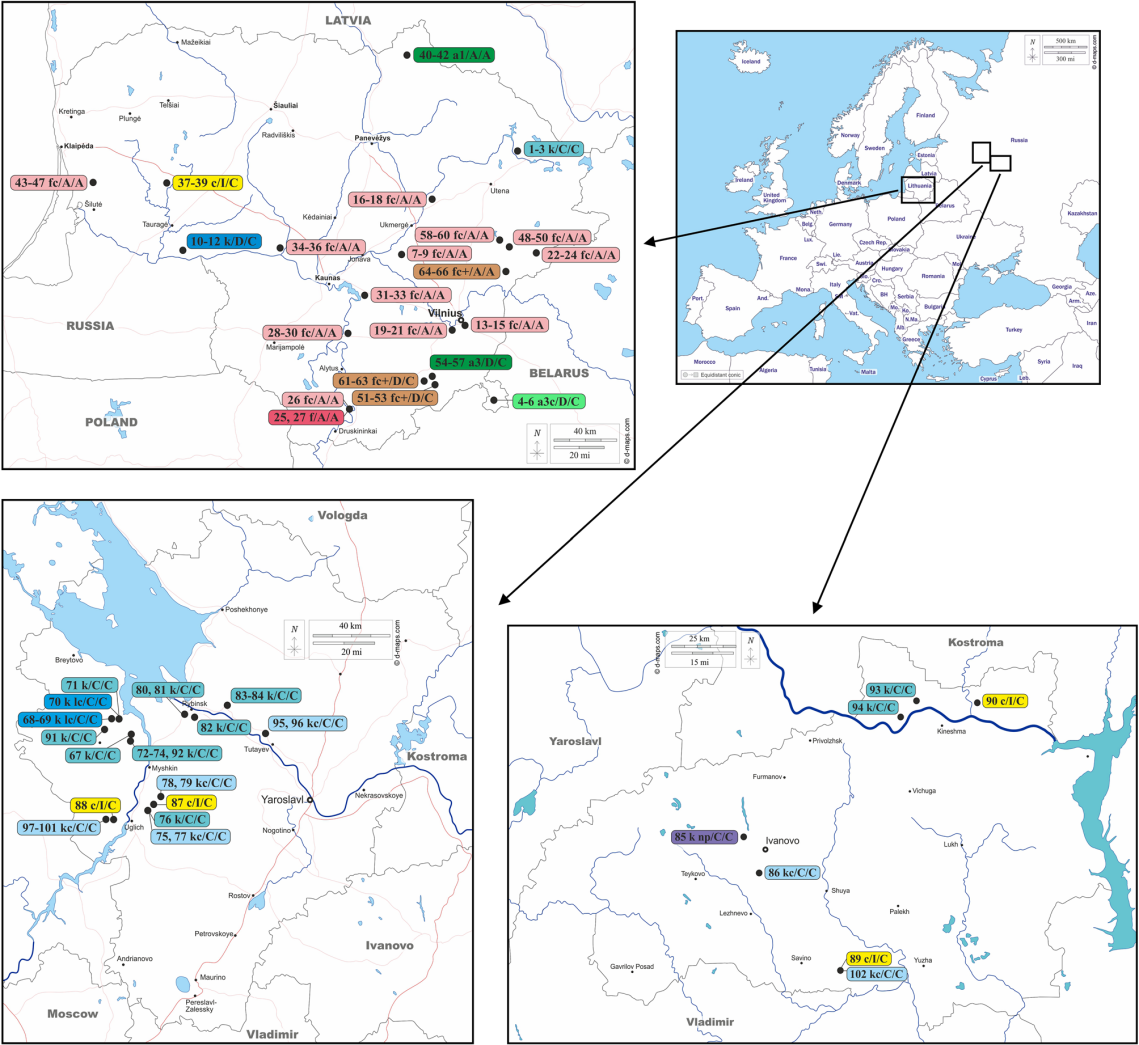
Study area and distribution of studied samples of *Ranunculus* section *Batrachium*. Sample marks include sample number or numbers, ITS info, rpl32-trnL and petL-psbE haplotypes according to Supplementary data: Table S1, for example, 34–36 fc/A/A means that samples 34–36 from Lithuania, the Dubysa River have hybrid ITS inherited from *R*. *circinatus* and *R*. *fluitans*, the rpl32-trnL haplotype A and petL-psbE haplotype A. (The basis of the maps were taken from d-maps.com Lithuania https://d-maps.com/carte.php?num_car=26445&lang=en; Ivanovo https://d-maps.com/carte.php?num_car=73493&lang=en; Yaroslavl https://d-maps.com/carte.php?num_car=93710&lang=en; Europe https://d-maps.com/carte.php?num_car=4576&lang=en).

In the present study, we want to clarify and compare the *Batrachium* diversity in the rivers of two areas of East Europe (Lithuania and Central European Russia) (Fig. 1) with similar glaciation history, environmental conditions and human activity inhabited by some similar morphotypes of river *Batrachium*. In our study we want to answer on the following questions: 1) Are species or hybrids more frequent in river habitats? 2) Do similar morphotypes have the same or different origin? 3) Which factors promote hybridization and success of hybrids? 4) Is hybridization bi- or unidirectional, and which mechanisms determine the crossing direction?

## Results

### Morphological variation and ecological preferences

Specimens collected in Lithuanian rivers can be assigned to 4 species and 3 putative hybrids (Table 1; Supplementary data: Table S1). Most of the specimens represent a large or medium-sized homophyllous plant type with elongated obconical capillary leaves and quite large flowers. Most of the collected plants were sterile bearing pedicels without fruits (just receptacles with decaying carpels) below the nodes with normally developed flowers and some flowers with 1—2 rugose or deformed petals. Only some of these plants able to produce ripe fruits after flowering.

**Table 1:** Comparison of selected morphological characters of studied species *Ranunculus aquatilis*, *R*. *circinatus*, *R*. *fluitans*, *R*. *kauffmannii* and found putative hybrids in Lithuania and Central European Russia.

Character	*R*. *aquatilis* (2 lineages)	*R*. *circinatus*	*R*. *fluitans*	*R*. *kauffmannii*	*R*. *aquatilis* × *R*. *circinatus*	*R*. *circinatus* × *R*. *fluitans*	*R*. *circinatus* × *R*. *fluitans* × ?	*R*. *circinatus* × *R*. *kauffmannii*
Plant size*	Medium-sized	Medium-sized	Large	Medium-sized to large	Medium-sized to large	Medium-sized to large, large	Medium-sized to large, large	Medium-sized to large
Floating leaves	Sometimes present (in one studied population sometimes present, in other absent)	Absent	Absent	Absent	Absent	Absent	Absent	Absent
Intermediate leaves	Frequently present (in studied population absent)	Absent	Absent	Absent	Absent	Absent	Absent	Absent
*Submersed leaves:*								
Number of the lamina divisions	≤ 6	≤ 6	≤ 4	≤ 6 (–7)	≤ 6	≤ 5 (–6)	≤ 5 (–6)	≤ 6
Number of the terminal segments	< 100	< 100	< 40	< 100 (–150)	< 100	< 60 (–90)	< 60 (–90)	< 100
Ratio of the leaf and corresponding internode in the upper part	Leaves equal to or slightly shorter than internodes	Leaves many times shorter than internodes	Leaves longer than or equal to internodes	Leaves equal to or slightly shorter than internodes	Leaves slightly to few times shorter than internodes	Leaves longer than or equal to or shorter than internodes	Leaves equal to or shorter than internodes	Leaves slightly to few times shorter than internodes
Shape of the lamina	Obconical to suborbicular	Circular to semicircular	Elongate-obconical	Obconical	Obconical to semicircular	Obconical	Obconical	Obconical to semicircular
Length of the lamina, mm	20–60	10–30	30–200 (–600)	60–100 (–150)	30–80	20–150	30–100 (–150)	30–80 (–100)
Length of the petiole, mm	5–30	0–5	5–50 (–200)	5–30	5–10 (–30)	5–20	10–30 (–50)	5–10 (–30)
Arrangement of the segments	Lying in different planes	Lying in one plane	Lying in different planes	Lying in different planes	Lying in different planes	Lying in different planes	Lying in different planes	Lying in different planes
Ratio of the middle and lateral lobes	Middle lobe slightly shorter than lateral lobes	Middle lobe equal to lateral lobes	Middle lobe more or less equal to lateral lobes (sometimes slightly shorter)	Middle lobe distinctly shorter than lateral lobes	Middle lobe slightly shorter than lateral lobes	Middle lobe slightly shorter than lateral lobes	Middle lobe shorter than lateral lobes	Middle lobe slightly shorter than lateral lobes
Diameter of the flowers, mm	10–20	15–20	15–30	10–15	15—20	15–25	15–20	10—20
Number of the petals	5	5	5–10	5	5	5, sometimes 6–8	5	5
Shape of the nectar-pits	Cup-shaped, circular, single (in one studied population cup-shaped, in other deeply lunate to almost circular)	Lunate, single	Pyriform, single	Lunate, single	Irregular cup-shaped to deeply lunate, single	Pyriform, elongated, single, rarely double	Cup-shaped to shortly pyriform, single	Lunate, single
Receptacles	Pubescent to hairy	Pubescent	Glabrous	Hairy	Pubescent to hairy	Glabrous to puberulent	Pubescent	Pubescent to hairy
Fruits	Present	Present	Present	Present	Absent	Absent	Absent	Absent

*Medium-sized plants (> 50 cm long), large plants (> 150 cm long).

We assigned fertile plants to *R*. *fluitans* Lam. (large plants with sparse leaf segments, 5 and more narrow petals, and glabrous receptacles) and to homophyllous *R*. *aquatilis* L. or *R*. *kauffmannii* Clerc (medium-sized plants with dense leaf segments, 5 quite wide petals, and pubescent receptacles) (Table 1). The two latter species can be distinguished from each other by the shape of their nectar pits (Table 1). The group of sterile plants consists of large plants with an intermediate number of leaf segments, rarely more than 5 wide petals and almost glabrous receptacles, and of medium-sized plants with dense leaf segments, 5 quite wide petals and pubescent receptacles. The first hybrid type represented by large plants can be identified based on morphological characters as a hybrid of *R*. *fluitans* with an unknown species, whereas the second medium-sized hybrid type as hybrids of *R*. *aquatilis* and/or *R*. *kauffmannii* with an unknown species. It is almost impossible to identify the second parent species in both cases based on morphological data alone. In only one locality we found the rigid-leaved species *R*. *circinatus* Sibth. (Fig. 1; Supplementary data: Table S1).

*Ranunculus aquatilis*, *R*. *fluitans*, *R*. *kauffmannii* and their putative hybrids occur in Lithuanian rivers in fast- to moderately flowing, relatively shallow water habitats (riffles and rapids). *R*. *fluitans* and its hybrid prefer deeper waters than *R*. *aquatilis*, *R*. *kauffmannii* and their putative hybrids. *Ranunculus circinatus* grows in still or slowly flowing deep water habitats (reaches) in these rivers, but occurs more frequently in lakes, ponds, and reservoirs.

Specimens in the Central European Russian rivers (Upper Volga region) can be assigned to 2 species and 1 putative hybrid (Table 1; Supplementary data: Table S1). Most of the collections belong to a medium-sized to a large homophyllous plant type with obconical capillary leaves and medium-sized flowers. Some of these plants were fertile and produced ripe fruits after flowering; the others were sterile, bearing numerous brownish elongated pedicels without fruits (just receptacles with decaying carpels) right below the nodes with normally developed flowers and some flowers with 1—2 rugose or deformed petals. We identified the first plant type as *R*. *kauffmannii* (Table 1), which is a characteristic taxon for rivers in the region. The second plant type belongs to a hybrid of *R*. *kauffmannii* and an unknown species, which is difficult to identify based on morphological characters. In some river habitats, we found fertile rigid-leaved *R*. *circinatus* (Fig. 1; Supplementary data: Table S1).

In Central European Russian rivers, *R*. *kauffmannii* occupies fast- to moderately flowing shallow water parts (riffles and rapids), and *R*. *circinatus* occurs only in still deep water parts (reaches and mouth parts) in these rivers. Like in Lithuania, *R*. *circinatus* is more common in lakes, ponds, and reservoirs of the region. Sterile hybrid plants tend to be more common toward the mouths of rivers as well as near human made habitats such as ponds, bridges and dams.

### Variation in the nuclear ITS region

**Figure 2:**
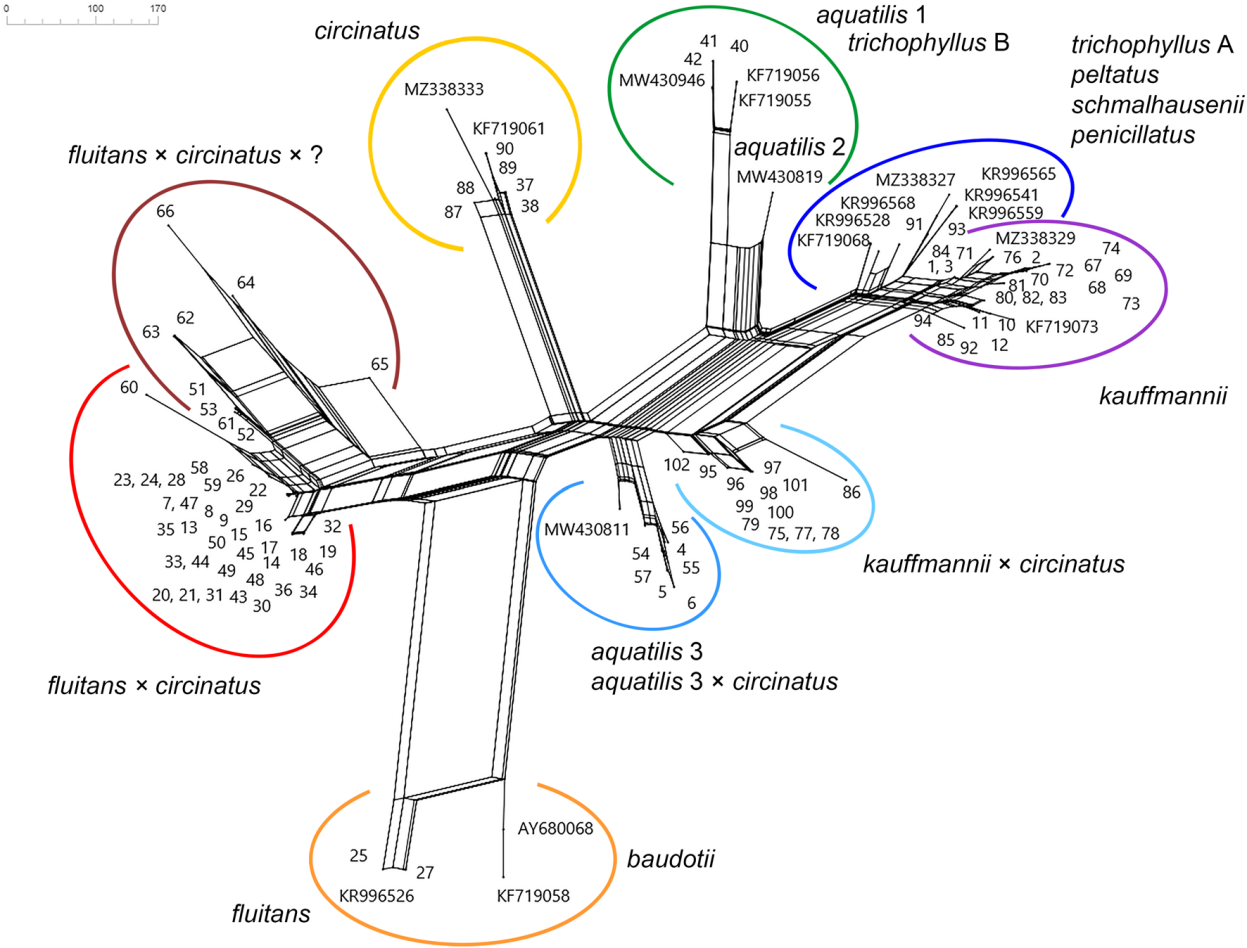
NeighborNet analysis of ITS variability within *Batrachium* studied samples and some additional species. Numbers correspond to sample numbers in Supplementary data: Table S1 and GenBank accession numbers. Hybrid names were given regarding the crossing direction: maternal species × paternal species.

Among our sequences we found only two *Ranunculus fluitans* samples with ITS sequences identical to those of the species from Poland (Fig. 2; Supplementary data: Table S2). This species and the closely related *R*. *baudotii* Godr. (namely its northern coastal lineage), shares an additional G at position 30, which allows to easily distinguish both from other taxa (Supplementary data: Table S2).

Samples of *R*. *circinatus* from Lithuania and Central European Russia show within-species variation and additive polymorphisms (Fig. 2; Supplementary data: Table S2). One sample from the easternmost locality in the Upper Volga region (Ivanovo Region) has ITS sequences identical to those of this species from Poland. The second ITS ribotype of this species differ by having G instead of A at position 86 and T instead of C at positions 86 and 180, and was known from the relic postglacial Plescheevo Lake in the Yaroslavl Region (namely MZ338333). The third ITS ribotype, which is closer to the first one and differs from it with G instead of A at position 86, was obtained from samples from westernmost localities in the Upper Volga region (Yaroslavl Region). Samples having ITS sequences with additive polymorphisms in this position occurred in one eastern locality in the Upper Volga region (Ivanovo Region) and also in Lithuania, but the latter samples has one more additive polymorphism in position 202 (Y instead of C) (Supplementary data: Table S2).

*Ranunculus aquatilis* was represented in Lithuania by two lineages (Fig. 2; Supplementary data: Table S2). Homophyllous *R*. *aquatilis* samples from northern Lithuania have almost identical ITS sequences with samples of this species lineage from Poland and Croatia, differing by one position 501 (C instead T), and identical to *R*. *trichophyllus* B sample from the Czech Republic. This lineage does not possess any additive polymorphisms. Samples of *R*. *aquatilis* from southeastern Lithuania which rarely develop laminar leaves similar to another *R*. *aquatilis* sample from the Czech Republic which possesses the additive combination of *R*. *trichophyllus* B and *R*. *circinatus*-like ITS. But our samples have more additive polymorphisms (at 14 positions) displaying influence rather of *R*. *peltatus* Schrank-like copy in addition to *R*. *circinatus*-like ITS (Supplementary data: Table S2).

*Ranunculus kauffmannii* ITS sequences represent 15 variants (Fig. 2; Supplementary data: Table S2). They display an additive polymorphism pattern at six sites and one five-base pair insertion in three sequences, except two samples without polymorphic sites. However, this pattern is not uniform and differs among the samples from just one locality. The six nucleotide positions where *R*. *kauffmannii* expresses additive polymorphisms are polymorphic in different *R*. *trichophyllus* Chaix lineages (Supplementary data: Table S2), which suggests that *R*. *kauffmannii* combines different ITS variants.

Most ITS sequences of sterile hybrid plants from Lithuania can be classified in three groups (Fig. 2; Supplementary data: Table S2).

The first group of samples comprises two variants which have additive polymorphisms at 21 positions: 20 single nucleotide polymorphisms and one single-base pair indel at alignment position 30, causing a shift in the overlapping ribotype sequences (Supplementary data: Table S2). Among the investigated samples, the additive polymorphism pattern is uniform at 20 positions, which are polymorphic in *R*. *circinatus* and *R*. *fluitans*, suggesting that they are the parental species. The remaining additive polymorphic positions could appear from some variation of ITS ribotypes of these parental species involved in the crossing.

Similar hybrid ITS sequences were found in second group of samples from three river localities in Lithuania (Grūda, Mera, Merkys) and are represented by two variants. They differ by additional additive polymorphisms at positions 64, 67, 72, 450, 465, 481 (Supplementary data: Table S2), which might be caused by additional hybridizations, most likely with some *R*. *aquatilis* lineages.

The third group of samples from southeastern Lithuania possess three ITS variants which has 16 additive single nucleotide polymorphic positions (14 constant) (Supplementary data: Table S2), which most likely differing the *R*. *aquatilis* lineage occurring in that part of country and *R*. *circinatus*.

All hybrid samples from Central European Russia comprise 4 variants of ITS sequences (Fig. 2; Supplementary data: Table S2). They have additive polymorphisms at 18 positions, and all are single nucleotide polymorphisms (Supplementary data: Table S2). Only 12 additive polymorphisms are constant among the samples, and they are polymorphic in *R*. *circinatus* and *R*. *kauffmannii*, suggesting that they are the parental species. The remaining additive polymorphic positions display the ITS sequence variation in *R*. *circinatus* and *R*. *kauffmannii*.

### Variation of the plastid DNA regions

**Figure 3:**
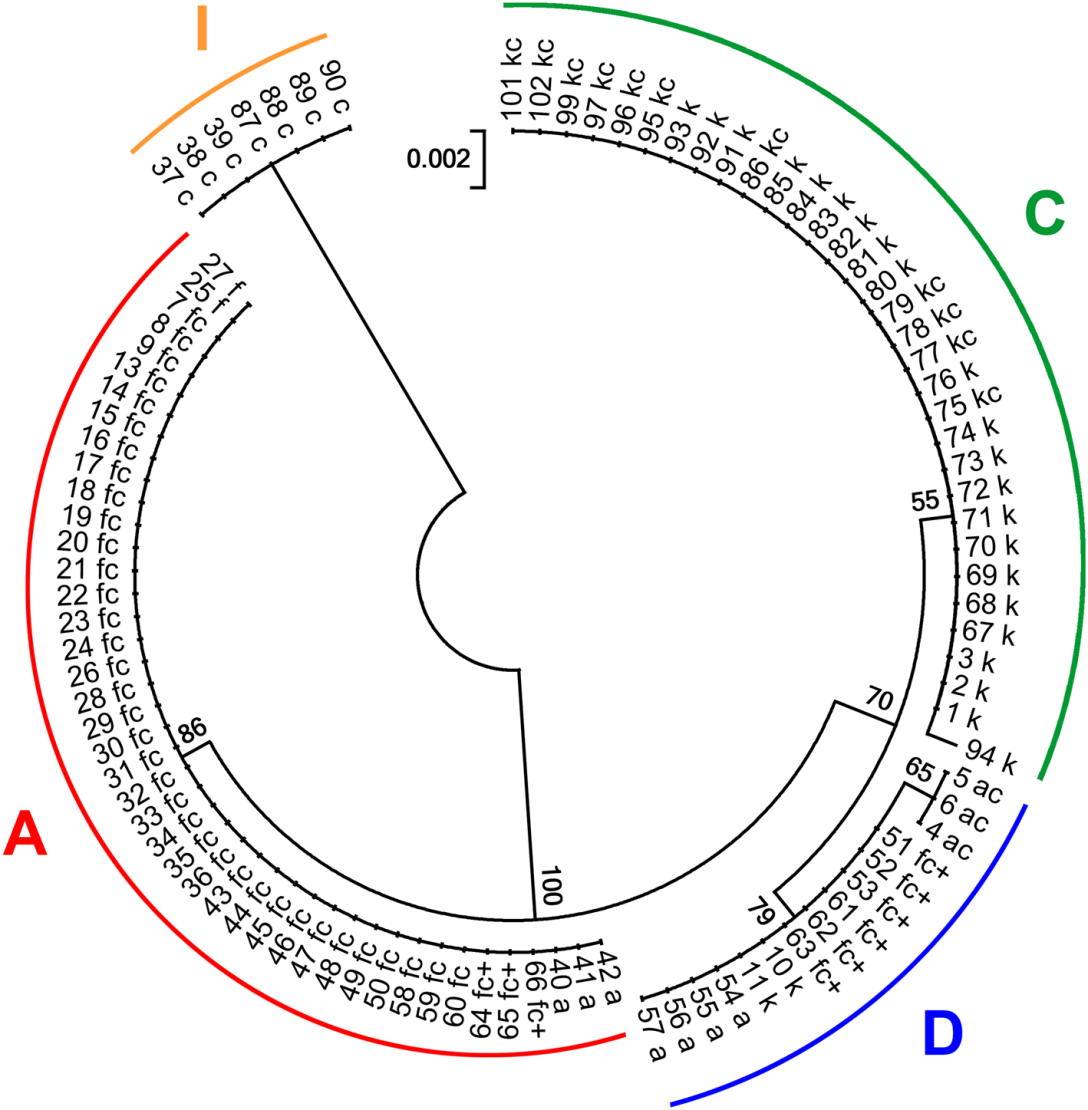
Phylogenetic relations within *Batrachium* studied samples based on the rpl32-trnL region. Numbers correspond to sample numbers in Supplementary data: Table S1. Haplotypes according to Bobrov et al. ^11^; see also Tables S1, S3. Bootstrap support values from 1000 replicates are shown next to the branches.

The studied individuals were divided into four clades according to variation in the rpl32-trnL intergenic spacer (Fig. 3, Supplementary data: Tables S1, S3). Clade A is represented by all samples of *R*. *aquatilis*, *R*. *fluitans*, the hybrid *R*. *circinatus* × *R*. *fluitans* and the complex hybrid closely related to the latter. Clade C includes all samples of *R*. *kauffmannii* and the hybrid *R*. *circinatus* × *R*. *kauffmannii* from Central European Russia and samples of *R*. *kauffmannii* from one locality in northeastern Lithuania. Closely related to clade C, clade D is represented by samples of *R*. *kauffmannii* from western Lithuania, the hybrid *R*. *aquatilis* × *R*. *circinatus* and the complex hybrid with participation of *R*. *circinatus* and *R*. *fluitans* from Lithuania. Within this clade, samples of the hybrid *R*. *aquatilis* × *R*. *circinatus* from one locality in southeastern Lithuania differ by one unique substitution. Clade I includes all samples of *R*. *circinatus* from Lithuania and Central European Russia. All described clades are represented by a single haplotype, except that clade D has an additional slightly deviating variant (Fig. 3).

**Figure 4:**
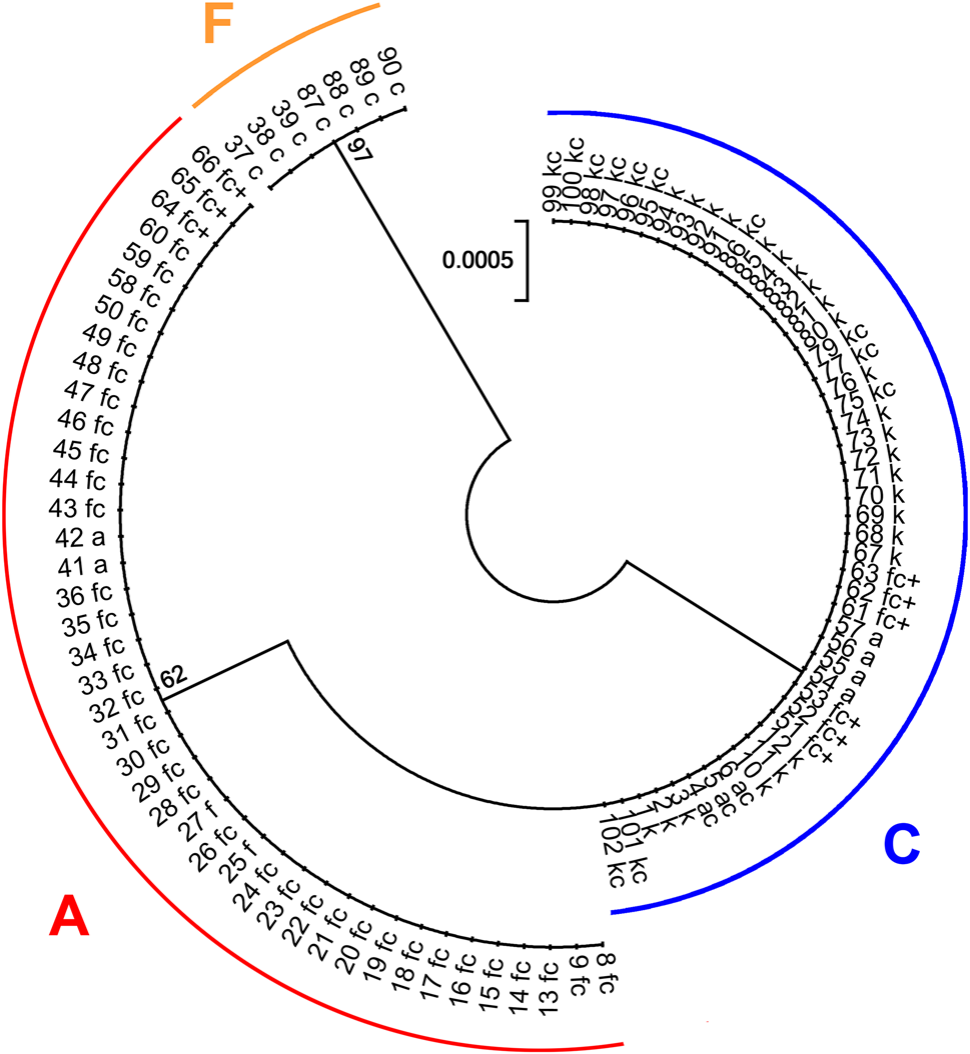
Phylogenetic relations within *Batrachium* studied samples based on the psbE-petL region. Numbers correspond to sample numbers in Supplementary data: Table S1. Haplotypes according to Bobrov et al. ^11^; see also Table S1, S4. Bootstrap support values from 1000 replicates are shown next to the branches.

According to variation in the petL-psbE intergenic spacer, all individuals are assigned to three clades (Fig. 4, Supplementary data: Tables S1, S4). Clade A is represented by all samples of *R*. *aquatilis*, *R*. *fluitans*, the hybrid *R*. *circinatus* × *R*. *fluitans* and the complex hybrid closely related to the latter. Clade C includes all samples of *R*. *kauffmannii*, the hybrids *R*. *aquatilis* × *R*. *circinatus*, *R*. *circinatus* × *R*. *kauffmannii* and the complex hybrid with participation of *R*. *circinatus* and *R*. *fluitans* from Lithuania and Central European Russia. Clade F is represented by all samples of *R*. *circinatus* from Lithuania and Central European Russia. All described clades include samples of a single haplotype (Fig. 4).

The plastid sequences of most hybrid samples are identical to those of *R*. *fluitans* in Lithuania and *R*. *kauffmannii* in Central European Russia, only some Lithuanian samples possess sequences characteristic of *R*. *aquatilis.* or western *R*. *kauffmannii* This indicates that these river species are a donor of chloroplasts, i.e., the maternal parents. Almost all discovered hybrids have *R*. *circinatus* as a pollen donor, i.e., paternal species. This demonstrates that the hybrids originated from unidirectional crossing.

## Discussion

### Variation, taxonomy and distribution

In the studied Lithuanian rivers, *R*. *aquatilis*, *R*. *fluitans*, *R*. *kauffmannii*, sterile hybrids *R*. *aquatilis* × *R*. *circinatus*, *R*. *circinatus* × *R*. *fluitans*, *R*. *circinatus* × *R*. *kauffmannii* and two complex hybrids *R*. *aquatilis* ? × *R*. *circinatus* × *R*. *fluitans* were found in fast-flowing habitats, and *R*. *circinatus* was recorded in still waters (Fig. 1). Such taxa composition differs from the previously known diversity recorded for this group in Lithuania^15,41–44^.

*Ranunculus aquatilis* from the Tatula River previously was regarded as *R*. *pseudofluitans* (Syme) Newbould ex Baker et Foggitt^41,43^. It differs from typical usually heterophyllous *R*. *aquatilis* by the absence of laminar leaves and closely resembles *R*. *trichophyllus* but it has somewhat intermediate deeply lunate to almost circular nectar pits. The studied plants have almost the same ITS characteristics as *R*. *aquatilis* samples from Croatia and Poland but share the plastid DNA of haplotypes A (haplotype of the *R*. *peltatus* group sensu Koutecký et al.^5^) together with all our *R*. *fluitans*, *R*. *circinatus* × *R*. *fluitans* and the single Polish *R*. *aquatilis* sample^11^. *Ranunculus aquatilis* from the Ūla River was also previosly reported as *R*. *pseudofluitans* or *R*. *penicillatus* (Dumort.) Bab. s.l. The Ūla River morphotype is closer to typical heterophyllous *R*. *aquatilis* by its ability to develop laminar leaves and by having cup-shaped nectar pits. These plants have similar ITS sequences to the *R*. *aquatilis* sample from the Czech Republic, i.e. additive polymorphism pattern due to the presence of *R*. *circinatus*-like ITS copy in combination with *R*. *trichophyllus* B or even *R*. *peltatus*-like copy. They shared the plastid DNA of the rpl32-trnL haplotype D and petL-psbE haplotype C (haplotype of the *R*. *trichophyllus* group sensu Koutecký et al.^5^) together with one Lithuanian *R*. *kauffmannii* according to the rpl32-trnL marker or with all our *R*. *trichophyllus*-like samples according to the petL-psbE marker as reported for some *R*. *aquatilis* lineages from Central Europe^5,11^.

In their study Koutecký et al.^5^ demonstrated that normally *R*. *aquatilis* has the additive ITS pattern and different variants of plastid haplotypes. Samples similar to our homophyllous plants (without polymorphic ITS) they^5^ treated as *R*. *trichophyllus* lineage B1 with plastid haplotype of the *R*. *peltatus* group (our A haplotypes). In our study all *R*. *trichophyllus*-like samples (our *R*. *kauffmannii* and additionally included different *R*. *trichophyllus* lineages) possess ITS ribotypes corresponding to the other *R*. *trichophyllus* lineage A by Koutecký et al.^5^ and shared plastid haplotypes of the *R*. *trichophyllus* group (our C and D haplotypes). It appears that these remote A (*R*. *trichophyllus*-like) and B (*R*. *aquatilis*-like) lineages of *R*. *trichophyllus* represents different species, as Koutecký et al.^5^ have also concluded. Therefore, it is clear that Central and West European *R*. *aquatilis*-*R*. *trichophyllus* B complex needs special attention as does as all *R*. *trichophyllus* A-like forms in the Holarctic context.

*Ranunculus circinatus* is a common species in Lithuania, but rarely occurs in flowing river habitats, therefore it was found in only one studied river locality. These samples have the same or very similar ITS and plastid marker sequences as the recently published Central European ones^5,30^.

The occurrence of *Ranunculus fluitans* in our study was confirmed in only one locality (Nemunas River) together with the hybrid *R*. *circinatus* × *R*. *fluitans*, despite the previous assumption that this species is common in Lithuanian rivers. According to molecular data, our samples of *R*. *fluitans* are identical to those recently published for Central Europe^5^ and Poland^30^.

*Ranunculus kauffmannii* was not previously reported in Lithuania. It had been regarded as *R*. *pseudofluitans* or *R*. *penicillatus* in Lithuania^41,43^. *Ranunculus kauffmannii* is represented by lineages that inherited the rpl32-trnL haplotype C characteristic for eastern European *R*. *trichophyllus*-like forms with capillary leaves only and the rpl32-trnL haplotype D characteristic for more western and northern *R*. *peltatus*-like and *R*. *trichophyllus*-like forms with capillary and laminar leaves. The first lineage of *R*. *kauffmannii* was found in northeastern Lithuania (Šventoji River), and the second type was found in the western (Viešvilė River) part.

We discovered a wide distribution of hybrids in Lithuanian rivers (see below). We found the hybrid *R*. *circinatus* × *R*. *fluitans* in almost all localities where *R*. *fluitans* was previously reported. In this study, this hybrid was observed in 12 of 21 studied rivers, in which it was found in 11 cases without *R*. *fluitans.* In only one locality these species and hybrid co-occurred. In all localities, the hybrid plants were sterile, and *R*. *fluitans* acted as a maternal parent. In all previous publications, the unusual features of local *R*. “*fluitans*”, its sterility and puberulent receptacles, were specifically noted^15,41,43,44^.

The closely related complex hybrid plants to *R*. *circinatus* × *R*. *fluitans* were found in the Grūda, Mera and Merkys rivers. Morphologically they differ from *R*. *circinatus* × *R*. *fluitans* by more dissected capillary leaves with finer segments, smaller flowers with wider petals and cup-shaped nectar-pits resembling river *R*. *aquatilis*-like or *R*. *kauffmannii*-like plants. These plants have ITS that clearly combine copies inherited from *R*. *circinatus* and *R*. *fluitans* with additional additive polymorphisms not characteristic of both species. These polymorphisms could appear from some additional hybridization. For plants from the Grūda and Merkys rivers, this assumption was supported by plastid markers belonging to rpl32-trnL haplotype D and petL-psbE haplotype C, which are characteristic of western and northern *R*. *peltatus*-like and *R*. *trichophyllus*-like forms with capillary and laminar leaves, and haplotypes shared with *R*. *aquatilis* distributed in the Merkys River basin (the Ūla River), which could have acted as a maternal parent. *Ranunculus aquatilis* has been pollinated by *R*. *circinatus* × *R*. *fluitans* which are unable to produce fruits but may contribute pollen. A similar pattern was recently found by Koutecký et al.^5^ for Central European plants and treated by them as tetraploid *R*. *penicillatus* B. Plants from the Mera River possess plastid markers of haplotypes A shared with *R*. *fluitans* as well as some lineages of *R*. *aquatilis* and *R*. *peltatus*. These hybrid plants have the ITS sequences clearly influenced by the *R*. *aquatilis* lineage (with *R*. *peltatus*-like copy), therefore it is most likely that *R*. *aquatilis* with haplotypes A was a maternal species in this hybrid too, as in the above mentioned example.

Another hybrid discovered in Lithuania is *R*. *aquatilis* × *R*. *circinatus*. These sterile plants occurred only in one locality in the southeastern part of Lithuania (Gauja River). They originated from the crossing of the *R*. *aquatilis* lineage which was found not far away, in the Ūla River of the Merkys River basin. Plants from the Gauja River are morphologically and genetically similar to plants from the Ūla River. But while having very similar hybrid ITS and plastid DNA, the former are sterile and the latter are fertile. The Ūla River *R*. *aquatilis* possess polymorphic hybrid ITS due to combination of *R*. *circinatus*-like and possibly *R*. *peltatus*-like copies, therefore they already inherited *R*. *circinatus*-like copy. Additional hybridization with *R*. *circinatus* gave more prominent peaks of this species on the ITS electrophoregrams from the Gauja River samples. *Ranunculus aquatilis* acted as a maternal parent in the formation of this hybrid. There is another explanation for the similarity of these plants. The Gauja River plants could originate from a crossing of *R*. *peltatus* and *R*. *circinatus* and gave the sterile triploid (2n = 3x = 24) hybrid. In a previous study^15^, we demonstrated that the Ūla River plants have 2n = 6x = 48, therefore they could evolve from this triploid hybrid in the result of the whole genome duplication. But we can get the pros and cons for this explanation only by counting chromosome or estimation genome size, as in the case of the hybrid plants from the Skroblus River^15^ of the same basin.

In previous morphological and ISSR analyses^41^ based on specimens from some of the same river localities, samples were split into two groups. This result corresponds well with the present data on morphology (Table 1) and molecular marker variation (Figs. 2, 3, 4, Supplementary data: Tables S1—S4). The first group consists of *R*. *fluitans*, *R*. *circinatus* × *R*. *fluitans* and the complex hybrid *R*. *aquatilis* ? × *R*. *circinatus* × *R*. *fluitans*, which are concentrated in the Neris River basin, and the second group consists of *R. aquatilis*, *R*. *aquatilis* × *R*. *circinatus* and the complex hybrid *R*. *aquatilis* ? × *R*. *circinatus* × *R*. *fluitans*, which are concentrated in the Merkys River basin and adjacent territory.

In the studied Central European Russian rivers, *Ranunuculus kauffmannii* and the sterile hybrid *R*. *circinatus* × *R*. *kauffmannii* were found in fast-flowing parts, while *R*. *circinatus* and the hybrid *R*. *circinatus* × *R*. *kauffmannii* were collected in still waters (Fig. 1). Such taxa composition differs slightly from the previously reported diversity for the group in the area^45,46^.

*Ranunculus circinatus* is a common species in Central European Russia, but avoids flowing waters, therefore in the studied rivers it was found in four localities. According to molecular data our samples of *R*. *circinatus* more variable than those recently published for Central Europe^5^ and Poland^30^.

*Ranunculus kauffmannii* is a common species in Central European Russia that is characteristic of fast-flowing river habitats. In this region, *R*. *kauffmannii* is represented by only one lineage that inherited the rpl32-trnL haplotype C characteristic for eastern European *R*. *trichophyllus*-like forms with capillary leaves only. No other variants have been found in the studied area. In the closely situated Ild and Sutka rivers, some samples contained a long copy of ITS, which has a duplication of CCCCA in position 401—405. These plants occur together with plants without such duplication in ITS in the same river and locality. In the studied rivers, *R*. *kauffmannii* was found in 12 localities.

We discovered a wide distribution of hybrids in rivers of the Upper Volga basin (see below). We found the hybrid *R*. *circinatus* × *R*. *kauffmannii* in only one locality of one river (Uleima River) together with *R*. *kauffmannii*, where *R*. *kauffmannii* and *R*. *circinatus* grew close to each other, upstream and downstream respectively. In two localities (Urdoma, Vostra rivers), only hybrids occurred. We also found hybrids with *R*. *circinatus* in two localities (Korozhechna and Shizhegda rivers). In all localities, the hybrid plants were sterile, and *R*. *kauffmannii* acted as a maternal parent. According to hybrid ITS variation hybrids in each locality share ITS copies of local or closely related populations of parental *R*. *circinatus* and *R*. *kauffmannii*. In the studied rivers, we have not found *R*. *circinatus* or its hybrids inherited an ITS ribotype identical to sample MZ338333 from relic postglacial Plescheevo Lake.

The hybrid *R*. *circinatus* × *R*. *kauffmannii* was previously reported from rivers in the Upper Volga region as *R*. *circinatus* × *R*. *trichophyllus*^45^. Moreover, according to the herbarium collection in the IBIW, a significant portion of river specimens identified as *R*. *trichophyllus* could actually be *R*. *circinatus* × *R*. *kauffmannii*, especially from the rivers where *R*. *circinatus* and *R*. *kauffmannii* co-occurred.

We may conclude that the *Batrachium* diversity differs in the rivers of Lithuania and Central European Russia despite similarity of morphotypes. In the former area, the West and Central European forms *R*. *aquatilis*, *R*. *fluitans* and their hybrids are dominant and represented by a larger number of taxa (4 species and 3 hybrids) whereas in the latter, the East European species *R*. *kauffmannii* and its hybrid are the only dominant forms with less total diversity (2 species and 1 hybrid). Althought both areas share *R*. *circinatus* and *R*. *kauffmannii*, the former is common and abundant in both areas, while *R*. *kauffmannii* become rare westward.

The rpl32-trnL region proved to be a highly variable marker with good resolution in *Batrachium*, therefore it is deservedly used for phylogenetic studies of the section^5,11,15^. We found that the petL-psbE region has significantly less resolution, and we do not recommend it for further studies although it was succesfully used in some previous works^11^.

### Rivers as “evolutionary incubator”

The important role played by hybridization in increasing the taxonomic diversity, speciation and evolution of *Batrachium* has been reported and discussed in many publications^3–13^. However, molecular tools for hybrid identification have been applied in few papers^4,5,10–13^. Among these papers, only three^4,5,30^ reported both the existence of hybrids and also their frequency among the studied samples. For example, Prančl et al.^4^, used flow cytometry and chromosome number data and evaluated 16 cytotypes of hybrid origin (seven identified and the rest unidentified) for Central Europe. In a subsequent study completed by genetic data and with extended data set, Koutecký et al.^5^ detected four more unidentified hybrids. Both studies determinated that not less than 15% of the individuals were hybrids. In Poland, Gebler et al.^30^ reported hybrid *R*. *circinatus* × *R*. *fluitans* for 10 rivers of 49 rivers studied (> 20%), and 13 this hybrid clones were found among 58 populations studied (> 22%).

We demonstrated that in Lithuanian rivers, among 66 studied *Batrachium* individuals, 18 represent species from six localities, and 48 represent hybrids from 16 localities; in only one locality species and its hybrid co-occurred. Therefore, hybrids make up 73% of individuals from 76% of localities, and only hybrids occupied 71% of localities.

In Central European Russian rivers, among 36 studied *Batrachium* individuals, 23 represent species from 16 localities, and 13 represent hybrids from six localities; in three localities, species and hybrids co-occurred. Therefore, hybrids make up 36% of individuals found in 32% of localities. In 16% of localities only hybrids were found.

These data are in accordance with the results by Prančl et al.^4^ and Koutecký et al.^5^, and in fact demonstrate a much wider distribution and larger role of *Batrachium* hybrids in river ecosystems. We should note that we summarized only sterile hybrids, while in rivers in fast-flowing habitats hexaploid *R*. *aquatilis* and tetraploid *R*. *kauffmannii* occur in Lithuania, and tetraploid *R*. *kauffmannii* ^47^ are the most common and abundant in the Upper Volga basin, which originated in the result of crossing of different species lineages.

The wide distribution and abundance of *Batrachium* hybrids and polyploids in the rivers of two distant areas suggest that rivers and streams play a role as “evolutionary incubators” for such newly arising hybrids and polyploids^4^.

We propose several explanations for this fact based on the studied rivers. First, rivers are favorable for hybrids and allopolyploids as they are “open environments”^18,48^, because in newly created habitats, which are frequently developing in river ecosystems, hybrids have a good chance to originate and survive. This is true for *Batrachium* diversity in Europe^1,4,5,12,13,16,30,49^. We have seen this also on the example of *Potamogeton* and *Stuckenia* in the rivers of Lithuania and the Upper Volga region, where hybrids exhibit a significant diversity and abundance^35,50,51^, as well as in other areas^21,33^.

The second source of hybridization occurs when species grow near the edges of their distribution range and occupy specific habitats representing extralimital or marginal populations which often show reduced competitive ability. In this case, they may be vulnerable to being lost to hybridization with more common and abundant local and tolerant species^18,20^, especially in response to ongoing climate change^52–55^. *Ranunculus fluitans* is growing in Lithuania near the eastern edge of its range^1^ and has an ecological optimum in permanently flowing waters of relatively large rivers with stable pebble bottoms in nonlimestone areas^3^. In Lithuania, it grows in the Nemunas River and most likely in the Neris River basin in a similar but less optimal environment^44^.

The next, Alahuhta et al.^62^ found that current climate (especially mean annual temperature) was the main environmental driver of aquatic plant species richness in Europe and North America. In the North-West of European Russia, which is situated almost between studied areas, the mean annual temperature began to exceed the climatic norm by 0.9 to 1.2 °C per decade since 1989^63^. Such a large increase of mean annual temperature may affect cold tolerant species^53,54^, i.e. river *R*. *fluitans* and *R*. *kauffmannii*. We have observed *R*. *kauffmannii* populations in several rivers in the Upper Volga region since late 1990s and can conclude that the number and area of populations has decreased more than two times. Climate change can promote the success of hybrids, not their origin, but rather their expansion.

The glaciation history of the landscapes is closely related to the previous two factors^21^. In postglacial areas of northern Europe, a number of sterile hybrids of aquatic vascular plants occur outside the modern ranges of parental species, e.g., *Nuphar*^56^, *Potamogeton*^57,58^ and *Stuckenia*^36,59^. The hybrids can survive better in postglacial areas compared to their parents since last glaciation by being more vigorous and adaptive. The studied rivers in Lithuania and the Upper Volga area were mostly covered with ice or were in the edge zone during the last Valdai (Würm, Weichselian) glaciation^60^. Therefore, the studied rivers predominantly run through a fluvioglacial landscape in the marginal zone of this glaciation. Wiegleb (pers. comm.) reported that the hybrid *R*. *circinatus* × *R*. *fluitans* is characteristic for glacial areas in Central Europe. There in the inflow and outflow regions of glacial lakes *R*. *circinatus* and *R*. *fluitans* can coexist despite different ecology. This was illustrated by Vollrath and Kohler^49^ in Bavaria and Gebler et al.^30^ in Poland in the Drawa River.

The last factor for hybridization is human impact^18,20^. All studied rivers are affected by pollution and eutrophication, the construction of dams and bridges, and the straightening of channels, which have severely changed the environmental characteristics of the rivers and destroyed the ecological barriers between species^50,61^. In West and Central Europe, the connection of formerly separate river catchments by canals, which occurred in most countries in the late 18th and throughout the nineteenth century, could served as a major trigger of hybridization. Such transformations of river ecosystems usually reduce the abundance of fast flowing water species (*R*. *fluitans*, *R*. *kauffmannii*) at the expense of still water species (*R*. *circinatus*). For example, Wiegleb [pers. comm.] reported that putative hybrid *R*. *circinatus* × *R*. *fluitans* occurs outside glacial deposits in highly modified water bodies in Great Britain, e.g., in the Stour River.

Therefore, we think that the wide distribution and abundance of *Batrachium* hybrids in the rivers of Lithuania and Central European Russia arose, first of all, due to the specificity of rivers as an open-type ecosystems and then were intensified by transformations of river ecosystems resulting from human activities. Evidently, the postglacial character of the studied river landscapes and ongoing climate change also have significant effect.

Moreover, the hybrid genomes provide greater plasticity of plants and, ultimately, the possibility of rapid adaptation to variable river conditions. Thus, hybrids are able to grow in fast and quiet currents in different bottom sediments, which ensures greater ecological and biological benefits in comparison to the parental species.

### Why only *Ranunculus circinatus* is a pollen donor?

Most hybrids of *R*. *aquatilis*, *R*. *fluitans* and *R*. *kauffmannii* had *R*. *circinatus* as a pollen donor; all hybrids originated from unidirectional crossing. The same pattern was found for Central European *R*. *circinatus* × *R*. *fluitans*^5,30^.

A similar case was reported by Prančl et al.^4^ for *R. peltatus* × *R. trichophyllus*, when unidirectional gene flow might be driven by a large difference in flower size: the large-flowered *R. peltatus* is more likely to be a pollen donor than the small-flowered *R. trichophyllus*. The small-flowered *Utricularia tenuicaulis* Miki served mostly as maternal species in hybridization with the large-flowered *U*. *macrorhiza* Leconte^64^. The same explanation could be applied in our case, but there was no difference between flower size in *R*. *circinatus* and *R*. *aquatilis* or *R*. *fluitans*, and the flowers of *R*. *circinatus* were slightly larger than the flowers of *R*. *kauffmannii* (Table 1). Moreover, all *Batrachium* species pollinated by insects and their hybrids can potentially originate in bidirectional crossing. Some additional factors should drive such a crossing direction.

We also suspect difference in the frequency of the parent species where more rare species are under strong introgression pressure by their more frequent neighbors^65–67^. In Lithuania, *R*. *circinatus* occurs more frequently and in more abundant populations than river *R*. *aquatilis* and *R*. *fluitans*. In the Upper Volga region, *R*. *kauffmannii* is as generally frequent and abundant species as *R*. *circinatus*, but in areas closely situated to the reservoirs of the Volga River cascade, the latter is clearly a more abundant species occupying large areas of the reservoir’s bays and shallow waters.

In Lithuania, the flowering period of *R*. *aquatilis*, *R*. *fluitans* and *R*. *circinatus* is usually similar. In the Upper Volga region, the flowering period of *R*. *kauffmannii* usually starts and finishes at least two weeks earlier (the beginning of June—the beginning of July) than *R*. *circinatus* (the middle of June to the beginning of August), and the flowering peaks of these species (the middle—the end of June and the end of June to first half of July, respectively) usually do not overlap each other (AB, EC observations). Therefore, *R*. *kauffmannii* is usually in full bloom and ready for pollination when *R*. *circinatus* only begins to flower and produce pollen, and not vice versa.

In addition, the asymmetric hybridization can be explained by self-compatibility mechanisms^68^, where a self-compatible species can be successfully crossed with pollen from a self-incompatible species, whereas the opposite configuration cannot succeed. In Lithuania, *R*. *aquatilis* and *R*. *circinatus* normally develop numerous fruits, but *R*. *fluitans* does this very rarely.

We have checked herbarium collections of *R.* *kauffmannii* and *R.* *circinatus* from the Upper Volga region in IBIW and found that almost all mature specimens of *R. kauffmannii* bear numerous fruits on almost all pedicels whereas only some mature specimens of *R. circinatus* have fruits on only some pedicels. Indirectly this may suggest that the former species is a self-compatible species and the latter is a self-incompatible species. Such asymmetric crossing was found for *Utricularia tenuicaulis* and *U. macrorhiza*^64^.

In the case of complex hybrids of *Ranunculus aquatilis* with *R*. *circinatus* × *R*. *fluitans*, more rare *R. aquatilis* has been pollinated by the more frequent and abundant *R*. *circinatus* × *R*. *fluitans* which is able to produce fertile pollen.

Therefore, different factors could determine the unidirectional crossing in different areas. Thus, frequency and abundance of populations in Lithuania with slightly more mild climate completed by the difference in flowering period and maybe a level of self-compatibility in the colder Upper Volga region could be the main explanations why almost always the rigid-leaved species *R*. *circinatus* acted as a pollen donor in case where parental species share common pollinators and have similar flower morphology.

### Descriptions of new taxa

The taxonomy of *Batrachium* is not resolved yet because diversity of this groups consists of diploid species, autopolyploids, allopolyploids, cryptic species, primary hybrids and introgressants^5^. To avoid this complex pattern we should first resolve and describe well-defined diploid and polyploid species and clearly evidenced primary hybrids, which will be necessary for further resolution of more complex forms and solving old nomenclatural confusions.

### *Ranunculus* × *redundans* A. A. Bobrov, Butkuvienė et Sinkevičienė nothosp. nov. (*R. circinatus* × *R. fluitans*)

#### Type

Lithuania, Varėna Distr., Nemunas River upstream of Merkinė, rapid, flow edge with weak current, 54.148938° N, 24.18015°, 22.06.2020, Butkuvienė J., Sinkevičienė Z. (holotype: BILAS92889 sheet 1, BILAS92890 sheet 2). As paratypes we can designated all the rest specimens of the hybrid listed in Supplementary data: Table S1. For image of the holotype see Supplementary data: Fig. S1.

Only genetically tested plants were selected as holotype and paratypes.

#### Diagnosis

Hybrid differs from *R*. *circinatus* by much longer and more flaccid capillary leaves with fewer terminal segments, flowers with narrower petals, and an almost glabrous or puberulent receptacle; from *R*. *fluitans* by shorter and more rigid leaves with more numerous terminal segments, flowers with wider petals, and puberulent receptacle; it is sterile, some flowers have rugose or deformed petals, pedicels are elongating and rapidly decaying just after flowering. Hybrid more resembles *R*. *fluitans* but hybrid plants are of dark green color not fresh to light green as is the species, and the hybrid usually develops short axillary side shoots in the late vegetation period, which are adnate to the stem or the adjacent petiole bearing small *R*. *circinatus*-like leaves.

Hybrid differs from morphologically similar forms such as *R*. *penicillatus* and *R*. *schmalhausenii* Luferov by absence of laminar and intermediate leaves with the middle lobe just slightly shorter than the lateral ones, fewer capillary leaf lamina divisions and number of segments, from *R*. *pseudofluitans* and *R*. *vertumnus* (C. D. K. Cook) Luferov by less number of capillary leaf lamina divisions and number of segments and almost glabrous receptacles.

#### Description

See Table 1.

#### Etymology

The taxon’s epithet refers to “niche equivalent” or “vicariant” because the hybrid closely resembles and actually mimics *R*. *fluitans*, and was previously regarded as *R*. *fluitans*.

#### Note

This hybrid was first reported from southern Germany by Vollrath, Kohler^46^ but without a binominal name. It was confirmed for Germany based on genetic data by Koutecký et al.^5^ and for Poland by Gebler et al.^30^. It is possible that this hybrid is not rare in the area where both parental species occur (West, Central and western East Europe). For example, Wiegleb [pers. comm.] reported such a hybrid from Great Britain (the Stour River).

Wiegleb et al.^1^ listed this taxon under *R*. *pseudofluitans* following the British tradition. However, the type specimen of *R*. *aquatilis* var. *pseudofluitans* Syme (lectotype, BM000057127) is not this taxon but resembles the hybrid of *R*. *fluitans* with *R*. *peltatus*-like or *R*. *trichophyllus*-like forms without laminar leaves.

### *Ranunculus* × *absconditus* A. A. Bobrov et Chemeris nothosp. nov. (*R. circinatus* × *R. kauffmannii*)

#### Type

Russia, Yaroslavl Reg., Tutaev Distr., near Vypolzovo village, Urdoma River, downstream bridge, 57.941523° N, 39.495951° E, 23.06.2013, Belyakov E. A. (holotype: IBIW71591; isotypes (currently at IBIW, to be distributed to several other herbaria): IBIW71592–71605). As paratypes we can designated all the rest specimens of the hybrid listed in Supplementary data: Table S1. For image of the holotype see Supplementary data: Fig. S2.

Only genetically tested plants were selected as holotype, isotypes and paratypes.

#### Diagnosis

Hybrid differs from *R*. *circinatus* by longer and more flaccid capillary leaves, and usually smaller flowers with narrower petals; from *R*. *kauffmannii* by shorter and more rigid leaves, and usually larger flowers with wider petals; it is sterile, some flowers can be underdeveloped with some rugose or deformed petals, pedicels are elongating and rapidly decaying just after flowering.

Hybrid differs from morphologically similar hybrid *R*. *circinatus* × *R*. *trichophyllus* by longer leaves and larger flowers, also it occurs only in river habitats or clearly connected to river in comparison to a wide range of stagnant water bodies characteristic of *R*. *circinatus* × *R*. *trichophyllus*.

#### Description

See Table 1.

#### Etymology

The taxon’s epithet refers to “hidden” because the hybrid resembles some stages of *R*. *kauffmannii* and some lineages of *R*. *trichophyllus* to which it was previously frequently but wrongly attributed, and it is difficult to recognize by morphological characters without molecular analysis.

#### Note

This hybrid was already reported without description and binominal name from the Upper Volga, European Russia, and Poland (see^1^). It is possible that this hybrid is not rare in the area where both parental species occur (mainly, boreal part of East Europe).

Wiegleb et al.^1^ listed the similar hybrid *R*. *circinatus* × *R*. *trichophyllus* from several countries in West, Central and East Europe, where it was recognized based on morphological characters. Taking into account the situation with cryptic lineages of *R*. *trichophyllus* found by Koutecký et al.^5^ and discussed above, all these records needs evaluation using molecular tools.

## Conclusion

The study and comparison of *Ranunculus* section *Batrachium* diversity in the rivers of Lithuania and Central European Russia, two areas of East Europe with similar historical backgrounds and human activity, based on a combination of molecular techniques with morphological analysis of herbarium collections and natural populations, allowed us to discover a wide distribution and abundance of hybrids between the river species *R*. *aquatilis*, *R*. *fluitans* and *R*. *kauffmannii* with the still water species *R*. *circinatus*. The *Batrachium* species and hybrid diversity is higher in the rivers of Lithuania (4 species and 3 hybrids vs. 2 and 1) and represented mainly by western *R*. *aquatilis*, *R*. *fluitans* and their hybrids whereas in Central European Russia, the East European species *R*. *kauffmannii* and its hybrid are the only dominant forms. Hybrids make up about 3/4 of the studied individuals from 3/4 of the studied river localities in Lithuania and 1/3 of the individuals found in 1/3 of the localities in Central European Russia. Such extensive hybridization in river *Batrachium* may have arisen due to the specificity of rivers as open-type ecosystems. It may have been intensified by the transformation of river ecosystems by human activities and the postglacial character of the studied landscapes combined with ongoing climate change. Almost all hybrids of *R*. *aquatilis*, *R*. *fluitans* and *R*. *kauffmannii* originated from unidirectional crossings in which *R*. *circinatus* acted as a pollen donor. Such crossings could be driven by higher frequency and abundance of *R*. *circinatus* populations as well as by some biological mechanisms. Two hybrids, *R*. *circinatus* × *R*. *fluitans* and *R*. *circinatus* × *R*. *kauffmannii*, were formally described. We found a hybrid which most likely originated from additional crossing between *R*. *aquatilis* and *R*. *circinatus* × *R*. *fluitans*, representing some kind of “recently evolved biotype” arising due to hybridization and polyploidization in addition to well-defined lineages.

These results show that hybridization contributes greatly to the evolution of *Batrachium* in particular as well as to other groups of aquatic vascular plants. Further extensive study of the genetic variation of *Ranunculus* section *Batrachium* on the Eurasian or even global scale is needed to reconstruct the whole picture of its diversification.

## Materials and methods

### Collection of material

*Batrachium* samples were collected from 21 river sites in Lithuania and 19 in Central European Russia (Upper Volga region) (Fig. 1). In each locality, 10—12 individuals were collected for preparing herbarium specimens and morphological study as well as fresh and silica gel dried samples for molecular analysis. Collection of plant material complied with relevant national, and international guidelines and legislation (according to Meilinger et al.^69^). Plants were collected in distant *Batrachium* patches (ca. 5 m). Co-occurring species were also registered. Voucher specimens are preserved in herbaria BILAS in Lithuania and IBIW in Russia. Locality details are presented in Supplementary data: Table S1. We collected 408 specimens, and 102 samples were included in the molecular study. Whenever possible, we sampled flowering and fruiting *Batrachium* plants that could be reliably checked for generative characters for taxa identification and evaluation of fertility or sterility of the individuals (mainly by presence or absence of fruits).

### DNA extraction, PCR amplification and sequencing

Fresh plant leaves, 80–100 mg, or, as an exception silica gel dried plant material, 10–15 mg, was used for DNA isolation, according to a modified CTAB method^70^. The sample tissue was ground to a fine powder using Mixer Mill 400 (Retsch) and 3-mm tungsten beads. Total genomic DNA was extracted using a DNeasy Plant Kit (Qiagen) following the manufacturer’s protocol. Analyses of the ribosomal DNA ITS region and the plastid rpl32-trnL and petL-psbE regions were performed. Primers ITS1 (50-TCCGTAGGTGAACCTGCGG-30) and ITS4 (5’-TCCTCCGCTTATTGATATGC-3’) were used for the amplification of the nuclear DNA region^71^. The PCR was performed as follows: initial denaturation at 94 °C for 2 min; 30 cycles of 94 °C for 1 min, 54 °C for 30 s, DNA synthesis at 72 °C for 2 min; ending with 72 °C for 5 min. The primers rpl32 (5’-AGTTCCAAAAAAACGTACTTC-3’) and trnL (UAG) (5’-CTGCTTCCTAAGAGCAGCGT-3’) and petL (5’-GTAGAAAACCGAAATAACTAGTTA-3’) and psbE (5’-TATCGAATACTGGTAATAATATCAGC-3’) were used for the amplification of the noncoding chloroplast DNA region^72^. PCR was performed as follows: initial denaturation at 80 °C for 5 min; 30 cycles of 95 °C for 1 min, primer annealing at 50 °C for 1 min, DNA synthesis followed by a ramp of 0.3 °C/s to 65 °C, and primer extension at 65 °C for 4 min; ending with 65 °C for 5 min. The preparation of amplified DNA fragments for sequencing was conducted as described earlier^73^. Briefly, PCR products were visualized in a 1% TAE-agarose gel, and the bands were cut out and purified using a GeneJET Gel Extraction Kit (Thermo Fisher Scientific Baltics, Lithuania). To check the concentration and purity of the purified PCR products, a NanoDrop One C spectrophotometer was used (Thermo Fisher Scientific, Waltham, MA, USA) or a MaxLife fluorimeter (Biolabmix, Novosibirsk, Russia). PCR products were sequenced in both directions employing the primers used for amplification. Direct sequencing was performed at the BaseClear B.V. (Leiden, The Netherlands) sequencing center or at the ABI PRISM 3500 Genetic Analyzer (Applied Biosystems, Foster, CA, USA) in IBIW RAS (Borok, Russia).

### Molecular data analyses

The consensus sequences from the forward and reverse strands were manually verified and adjusted using Sequencher 5.4.6 software (Gene Codes Corp. 2016, USA). Alignments of sequences of all regions were conducted manually using BioEdit 7.2.5 software^74^. Additive nucleotide polymorphisms were examined by comparing the two strands to ensure their consistency and coded using IUPAC nucleotide ambiguity codes.

The ITS data were visualized as a phylogenetic network using SplitsTree5 v.5.0.0 alpha software^75^. We applied a NeighborNet method^76^ to obtain a distance matrix with distances computed with the Hamming Distances Ambig States algorithm, handling ambiguous states as Average States^77^. A splits network was obtained by application of the Splits Network Algorithm^78^.

Maximum likelihood analysis and phylogram reconstructions for rpl32-trnL and petL-psbE plastid markers were performed in MEGA 11.0^79^. We estimated substitution models separately for each plastid marker dataset. We found that the best model is the Tamura 3-parameter model with a uniform rate. Bootstrap values were estimated with 1000 bootstrap samples. Gaps were treated as missing data. Our alignments could be retrieved from GenBank as a PopSet (accession numbers are given in Supplementary data: Table S1). In the ITS analysis, we included some ITS sequences of potential taxa *Ranunculus aquatilis*, *R*. *baudotii*, *R*. *circinatus*, *R*. *fluitans*, *R*. *kauffmannii*, *R*. *peltatus*, *R*. *penicillatus*, *R*. *schmalhausenii*, *R*. *trichophyllus* available from GenBank (accession numbers are indicated in Fig. 2, Supplementary data: Table S2) for most of which we have seen voucher herbarium specimens in addition to our sequences for checking our species and hybrid identifications. The designation of the rpl32-trnL and petL-psbE haplotypes was given according to Bobrov et al.^11^.

### Morphological study

We examined the morphological characters of collected *Batrachium* taxa on the herbarium specimens preserved in BILAS and IBIW as well as in available virtual herbaria (LE, MHA, MW) and online sources (GBIF). We evaluated fertility of specimens based on presence of ripe fruits after flowering or their absence where only numerous brownish elongated pedicels without fruits were present.

## Supplementary Information

Supplementary Information.

## Data Availability

All data used in the manuscript are freely available. The datasets generated and analysed during the current study are available in the NCBI GenBank repository, accession numbers: OM692092-OM692192, OM721106-OM721204, OM721205-OM721303. Herbarium specimens are stored in BILAS and IBIW: holotype of *Ranunculus* × *redundans* (*R*. *circinatus* × *R*. *fluitans*) preserved in BILAS (Nos. 92889, 92,890) and holotype of *Ranunculus* × *absconditus* (*R*. *circinatus* × *R*. *kauffmannii*) preserved in IBIW (No. 71591).
