# Supplementary material for: Extensive hybridization in Ranunculus section Batrachium (Ranunculaceae) in rivers of two postglacial landscapes of East Europe
Source: Sci Rep. 2022 Jul 15;12:12088. doi: 10.1038/s41598-022-16224-0 (PMC9287324; doi:10.1038/s41598-022-16224-0)
Supplement: Supplementary file 1 — Supplementary Information. [file 41598_2022_16224_MOESM1_ESM.pdf]

Supplementary data

Table S1. Number, taxon name, ITS sequence determination, cpDNA sequence haplotypes (according to Bobrov et al., 2015) of the studied samples of *Batrachium*.
Hybrid names were given regarding the crossing direction: maternal species × paternal species.
ITS sequence determination: a — *aquatilis*, c — *circinatus*, f — *fluitans*, k — *kauffmannii*, k lc — *kauffmannii* long copy, k np — *kauffmannii* no polymorphisms, ? — unidentifiable by ITS species.
Haplotypes of the rpl32-trnL sequences (according to Bobrov et al., 2015): A — *fluitans* + *aquatilis* 1, 2 + *peltatus* 1 (western European), C — *trichophyllus* 2 + *kauffmannii* (eastern European), D — *trichophyllus* 1 + *aquatilis* 3 + *peltatus* 2 + *schmalhausenii* 1 (western and northern European), I — *circinatus* (rigid-leaved).
Haplotypes of the petL-psbE sequences (according to Bobrov et al., 2015): A — *fluitans* + *aquatilis* 1, 2 + *peltatus* 1 (western European), C — *trichophyllus* 1, 2, 5 + *confervoides* + *kauffmannii* + *aquatilis* 3 + *peltatus* 2 + *schmalhausenii* 1 (European), F — *circinatus* + *rionii* (rigid-leaved).

Reference:

Bobrov, A. A., Zalewska-Gałosz, J., Jopek, M. & Movergoz, E. A. *Ranunculus schmalhausenii* (section *Batrachium*, Ranunculaceae), a neglected water crowfoot endemic to Fennoscandia — a case of rapid hybrid speciation in postglacial environment of North Europe. *Phytotaxa*, 233, 101-138 (2015). https://doi.org/10.11646/phytotaxa.233.2.1

No.	Taxon	ITS	rpl32-trnL haplotype	petL-psbE haplotype	Locality	Fertility (presence of fruits)
1	*kauffmannii*	k<br>OM692103	C<br>OM721217	C<br>OM721117	Zarasai Distr., Antalieptė, Šventoji River, rapid, 55.65944° N, 25.864468° E, 17.06.2020, Butkuvienė J. (Šventoji 8) BILAS92913	fertile
2	*kauffmannii*	k<br>OM692104	C<br>OM721218	C<br>OM721118	Zarasai Distr., Antalieptė, Šventoji River, rapid, 55.65944° N, 25.864468° E, 17.06.2020, Butkuvienė J. (Šventoji 12) BILAS92917	fertile
3	*kauffmannii*	k<br>OM692105	C<br>OM721219	C<br>OM721119	Zarasai Distr., Antalieptė, Šventoji River, rapid, 55.65944° N, 25.864468° E, 17.06.2020, Butkuvienė J. (Šventoji 11) BILAS92916	fertile

No.	Taxon	ITS	rpl32-trnL haplotype	petL-psbE haplotype	Locality	Fertility (presence of fruits)
4	*aquatilis* 3+ × *circinatus*	a3 × c<br>OM692173	D<br>OM721286	C<br>OM721187	Šalčininkai Distr., Kalviai, Gauja River, rapid, 54.17965° N, 25.657883° E, 15.07.2020, Butkuvienė J. (Gauja 2) BILAS92919	sterile
5	*aquatilis* 3+ × *circinatus*	a3 × c<br>OM692174	D<br>OM721287	C<br>OM721188	Šalčininkai Distr., Kalviai, Gauja River, rapid, 54.17965° N, 25.657883° E, 15.07.2020, Butkuvienė J. (Gauja 3) BILAS92920	sterile
6	*aquatilis* 3+ × *circinatus*	a3 × c<br>OM692175	D<br>OM721288	C<br>OM721189	Šalčininkai Distr., Kalviai, Gauja River, rapid, 54.17965° N, 25.657883° E, 15.07.2020, Butkuvienė J. (Gauja 10) BILAS92927	sterile
7	*fluitans* × *circinatus*	f × c<br>OM692129	A<br>OM721242	A<br>-	Širvintai Distr., Apeikiškiai, Širvinta River, rapid, 55.089917° N, 24.591917° E, 17.06.2020, Butkuvienė J. (Širvinta 7) BILAS92852	sterile
8	*fluitans* × *circinatus*	f × c<br>OM692130	A<br>OM721243	A<br>OM721143	Širvintai Distr., Apeikiškiai, Širvinta River, rapid, 55.089917° N, 24.591917° E, 17.06.2020, Butkuvienė J. (Širvinta 8) BILAS92853	sterile
9	*fluitans* × *circinatus*	f × c<br>OM692131	A<br>OM721244	A<br>OM721144	Širvintai Distr., Apeikiškiai, Širvinta River, rapid, 55.089917° N, 24.591917° E, 17.06.2020, Butkuvienė J. (Širvinta 11) BILAS92856	sterile
10	*kauffmannii*	k<br>OM692106	D<br>OM721220	C<br>OM721120	Jurbarkas Distr., Karšuva forest, Viešvilė River, weak current, 55.086653° N, 22.406862° E, 08.07.2020, Sinkevičienė Z. (Viešvilė 9) BILAS92938	fertile

No.	Taxon	ITS	rpl32-trnL haplotype	petL-psbE haplotype	Locality	Fertility (presence of fruits)
11	*kauffmannii*	k OM692107	D OM721221	C OM721121	Jurbarkas Distr., Karšuva forest, Viešvilė River, weak current, 55.086653° N, 22.406862° E, 08.07.2020, Sinkevičienė Z. (Viešvilė 10) BILAS92939	fertile
12	*kauffmannii*	k OM692108	-	C OM721122	Jurbarkas Distr., Karšuva forest, Viešvilė River, weak current, 55.086653° N, 22.406862° E, 08.07.2020, Sinkevičienė Z. (Viešvilė 11) BILAS92940	fertile
13	*fluitans* × *circinatus*	f × c OM692132	A OM721245	A OM721145	Vilnius Distr., Vilnius city, Vilnia River, rapid, 54.678017° N, 25.321883° E, 15.06.2020, Butkuvienė J. (Vilnia 2) BILAS92813	sterile
14	*fluitans* × *circinatus*	f × c OM692133	A OM721246	A OM721146	Vilnius Distr., Vilnius city, Vilnia River, rapid, 54.678017° N, 25.321883° E, 15.06.2020, Butkuvienė J. (Vilnia 6) BILAS92817	sterile
15	*fluitans* × *circinatus*	f × c OM692134	A OM721247	A OM721147	Vilnius Distr., Vilnius city, Vilnia River, rapid, 54.678017° N, 25.321883° E, 15.06.2020, Butkuvienė J. (Vilnia 10) BILAS92821	sterile
16	*fluitans* × *circinatus*	f × c OM692135	A OM721248	A OM721148	Anykščiai Distr., Pavirinčiai, Virinta River, rapid, 55.433233° N, 25.055017° E, 08.06.2020, Butkuvienė J. (Virinta 1) BILAS92858	sterile
17	*fluitans* × *circinatus*	f × c OM692136	A OM721249	A OM721149	Anykščiai Distr., Pavirinčiai, Virinta River, rapid, 55.433233° N, 25.055017° E, 08.06.2020, Butkuvienė J. (Virinta 4) BILAS92861	sterile

No.	Taxon	ITS	rpl32-trnL haplotype	petL-psbE haplotype	Locality	Fertility (presence of fruits)
18	*fluitans* × *circinatus*	f × c OM692137	A OM721250	A OM721150	Anykščiai Distr., Pavirinčiai, Virinta River, rapid, 55.433233° N, 25.055017° E, 08.06.2020, Butkuvienė J. (Virinta 12) BILAS92869	sterile
19	*fluitans* × *circinatus*	f × c OM692138	A OM721251	A OM721151	Vilnius Distr., Vokė River by Trakų Vokė, rapid, 54.627183° N, 25.120333° E, 10.06.2020, Butkuvienė J. (Vokė 2) BILAS92801	sterile
20	*fluitans* × *circinatus*	f × c OM692139	A OM721252	A OM721152	Vilnius Distr., Vokė River by Trakų Vokė, rapid, 54.627183° N, 25.120333° E, 10.06.2020, Butkuvienė J. (Vokė 3) BILAS92802	sterile
21	*fluitans* × *circinatus*	f × c OM692140	A OM721253	A OM721153	Vilnius Distr., Vokė River by Trakų Vokė, rapid, 54.627183° N, 25.120333° E, 10.06.2020, Butkuvienė J. (Vokė 7) BILAS92806	sterile
22	*fluitans* × *circinatus*	f × c OM692141	A OM721254	A OM721154	Švenčionys Distr., Žeimena River downstream of Jaunadaris, rapid, 55.08375° N, 25.92775° E, 26.06.2020, Butkuvienė J. (Žeimena 2) BILAS92789	sterile
23	*fluitans* × *circinatus*	f × c OM692142	A OM721255	A OM721155	Švenčionys Distr., Žeimena River downstream of Jaunadaris, rapid, 55.08375° N, 25.92775° E, 26.06.2020, Butkuvienė J. (Žeimena 8) BILAS92795	sterile
24	*fluitans* × *circinatus*	f × c OM692143	A OM721256	A OM721156	Švenčionys Distr., Žeimena River downstream of Jaunadaris, rapid, 55.08375° N, 25.92775° E, 26.06.2020, Butkuvienė J. (Žeimena 10) BILAS92797	sterile
25	*fluitans*	f OM692092	A OM721303	A OM721106	Varėna Distr., Nemunas River upstream of Merkinė, rapid, 54.148938° N, 24.18015° E, 22.06.2020, Butkuvienė J., Sinkevičienė Z. (Nemunas 2) BILAS92883	?

No.	Taxon	ITS	rpl32-trnL haplotype	petL-psbE haplotype	Locality	Fertility (presence of fruits)
26	*fluitans* × *circinatus*	f × c OM692128	A OM721241	A OM721142	Varėna Distr., Nemunas River upstream of Merkinė, weak current, 54.148938° N, 24.18015°, 22.06.2020, Butkuvienė J., Sinkevičienė Z. (Nemunas 8) BILAS92889, 92890	sterile
27	*fluitans*	f OM692093	A OM721206	A OM721107	Varėna Distr., Nemunas River upstream of Merkinė, rapid, 54.148938° N, 24.18015°, 22.06.2020, Butkuvienė J., Sinkevičienė Z. (Nemunas 12) BILAS92893	?
28	*fluitans* × *circinatus*	f × c OM692144	A OM721257	A OM721158	Prienai Distr., Voseliūnai, Verknė River, rapid, 54.59645° N, 24.098283° E, 11.06.2020, Sinkevičienė Z. (Verknė 5) BILAS92827	sterile
29	*fluitans* × *circinatus*	f × c OM692145	A OM721258	A OM721159	Prienai Distr., Voseliūnai, Verknė River, rapid, 54.59645° N, 24.098283° E, 11.06.2020, Sinkevičienė Z. (Verknė 7) BILAS92829	sterile
30	*fluitans* × *circinatus*	f × c OM692146	A OM721259	A OM721160	Prienai Distr., Voseliūnai, Verknė River, rapid, 54.59645° N, 24.098283° E, 11.06.2020, Sinkevičienė Z. (Verknė 10) BILAS92832	sterile
31	*fluitans* × *circinatus*	f × c OM692147	A OM721260	A OM721161	Kaišiadorys Distr., Leliušiai, Strėva River, rapid, 54.816067° N, 24.29055° E, 15.06.2020, Butkuvienė J. (Strėva 1) BILAS92834	sterile
32	*fluitans* × *circinatus*	f × c OM692148	A OM721261	A OM721162	Kaišiadorys Distr., Leliušiai, Strėva River, rapid, 54.816067° N, 24.29055° E, 15.06.2020, Butkuvienė J. (Strėva 9) BILAS92842	sterile
33	*fluitans* × *circinatus*	f × c OM692149	A OM721262	A OM721163	Kaišiadorys Distr., Leliušiai, Strėva River, rapid, 54.816067° N, 24.29055° E, 15.06.2020, Butkuvienė J. (Strėva 12) BILAS92845	sterile

No.	Taxon	ITS	rpl32-trnL haplotype	petL-psbE haplotype	Locality	Fertility (presence of fruits)
34	*fluitans* × *circinatus*	f × c OM692150	A OM721263	A OM721164	Jurbarkas Distr., Seredžius, Dubysa River, rapid, 55.084267° N, 23.4345° E, 08.07.2020, Sinkevičienė Z. (Dubysa 2) BILAS92871	sterile
35	*fluitans* × *circinatus*	f × c OM692151	A OM721264	A OM721165	Jurbarkas Distr., Seredžius, Dubysa River, rapid, 55.084267° N, 23.4345° E, 08.07.2020, Sinkevičienė Z. (Dubysa 4) BILAS92873	sterile
36	*fluitans* × *circinatus*	f × c OM692152	A OM721265	A OM721166	Jurbarkas Distr., Seredžius, Dubysa River, rapid, 55.084267° N, 23.4345° E, 08.07.2020, Sinkevičienė Z. (Dubysa 7) BILAS92876	sterile
37	*circinatus*	c OM692094	I OM721207	C OM721108	Šilalė Distr., Indija, Akmena River, rapid, 55.446967° N, 22.232667° E, 13.07.2020, Butkuvienė J. (Akmena 4) BILAS92993	?
38	*circinatus*	c OM692095	I OM721208	C OM721109	Šilalė Distr., Indija, Akmena River, rapid, 55.446967° N, 22.232667° E, 13.07.2020, Butkuvienė J. (Akmena 10) BILAS92999	?
39	*circinatus*	-	I OM721209	C OM721110	Šilalė Distr., Indija, Akmena River, rapid, 55.446967° N, 22.232667° E, 13.07.2020, Butkuvienė J. (Akmena 11) BILAS93000	?
40	*aquatilis* 1 (cf. *trichophyllus* B)	a1 OM692100	A OM721214	-	Biržai Distr., Kirdonys, Tatula River, rapid, 56.16955° N, 24.5771° E, 07.07.2020, Butkuvienė J. (Tatula 1) BILAS92978	fertile
41	*aquatilis* 1 (cf. *trichophyllus* B)	a1 OM692101	A OM721215	A OM721115	Biržai Distr., Kirdonys, Tatula River, rapid, 56.16955° N, 24.5771° E, 07.07.2020, Butkuvienė J. (Tatula 5) BILAS92982	fertile

No.	Taxon	ITS	rpl32-trnL haplotype	petL-psbE haplotype	Locality	Fertility (presence of fruits)
42	*aquatilis* 1 (cf. *trichophyllus* B)	a1 OM692102	A OM721216	A OM721116	Biržai Distr., Kirdonys, Tatula River, rapid, 56.16955° N, 24.5771° E, 07.07.2020, Butkuvienė J. (Tatula 12) BILAS92982	fertile
43	*fluitans* × *circinatus*	f × c OM692153	A OM721266	A OM721167	Šilutė Distr., Miestaliai, Tenenys River, rapid, 55.429067° N, 21.4972° E, 13.07.2020, Butkuvienė J. (Tenenys 1) BILAS92984	sterile
44	*fluitans* × *circinatus*	f × c OM692154	A OM721267	A OM721168	Šilutė Distr., Miestaliai, Tenenys River, rapid, 55.429067° N, 21.4972° E, 13.07.2020, Butkuvienė J. (Tenenys 3) BILAS92896	sterile
45	*fluitans* × *circinatus*	f × c OM692155	A OM721268	A OM721169	Šilutė Distr., Miestaliai, Tenenys River, rapid, 55.429067° N, 21.4972° E, 13.07.2020, Butkuvienė J. (Tenenys 6) BILAS92899	sterile
46	*fluitans* × *circinatus*	f × c OM692156	A OM721269	A OM721170	Šilutė Distr., Miestaliai, Tenenys River, rapid, 55.429067° N, 21.4972° E, 13.07.2020, Butkuvienė J. (Tenenys 8) BILAS92901	sterile
47	*fluitans* × *circinatus*	f × c OM692157	A OM721270	A OM721171	Šilutė Distr., Miestaliai, Tenenys River, rapid, 55.429067° N, 21.4972° E, 13.07.2020, Butkuvienė J. (Tenenys 10) BILAS92903	sterile
48	*fluitans* × *circinatus*	f × c OM692158	A OM721271	A OM721172	Švenčionys Distr., Šeškuškė II, Lakaja River, rapid, 55.124033° N, 25.8988° E, 26.06.2020, Butkuvienė J. (Lakaja 1) BILAS92764	sterile
49	*fluitans* × *circinatus*	f × c OM692159	A OM721272	A OM721173	Švenčionys Distr., Šeškuškė II, Lakaja River, rapid, 55.124033° N, 25.8988° E, 26.06.2020, Butkuvienė J. (Lakaja 10) BILAS92772	sterile

No.	Taxon	ITS	rpl32-trnL haplotype	petL-psbE haplotype	Locality	Fertility (presence of fruits)
50	*fluitans* × *circinatus*	f × c OM692160	A OM721273	A OM721174	Švenčionys Distr., Šeškuškė II , Lakaja River, rapid, 55.124033° N, 25.8988° E, 26.06.2020, Butkuvienė J. (Lakaja 11) BILAS92773	sterile
51	"*penicillatus*" 2 (*aquatilis* 3+ × (*circinatus* × *fluitans*))	f × c × ? OM6921667	D OM721280	C OM721181	Varėna Distr., Marcinkonys forest, Grūda River, downstream of the bridge, rapid, 54.093517° N, 24.376067° E, 22.06.2020, Butkuvienė J. (Grūda 3) BILAS92956	sterile
52	"*penicillatus*" 2 (*aquatilis* 3+ × (*circinatus* × *fluitans*))	f × c × ? OM692168	D OM721281	C OM721182	Varėna Distr., Marcinkonys forest, Grūda River, downstream of the bridge, rapid, 54.093517° N, 24.376067° E, 22.06.2020, Butkuvienė J. (Grūda 7) BILAS92960	sterile
53	"*penicillatus*" 2 (*aquatilis* 3+ × (*circinatus* × *fluitans*))	f × c × ? OM692169	D OM721282	C OM721183	Varėna Distr., Marcinkonys forest, Grūda River, downstream of the bridge, rapid, 54.093517° N, 24.376067° E, 22.06.2020, Butkuvienė J. (Grūda 12) BILAS92965	sterile
54	*aquatilis* 3+ (with *circinatus*- and *peltatus*-like copies)	a3 OM692176	D OM721289	C OM721190	Varėna Distr., Trakiškės forest between Mančiagirė and Žiūrai villages, Ūla River, rapid, 54.143217° N, 24.441217° E, 22.06.2020, Butkuvienė J. (Ūla 5) BILAS92946	fertile
55	*aquatilis* 3+ (with *circinatus*- and *peltatus*-like copies)	a3 OM692177	D OM721290	C OM721192	Varėna Distr., Trakiškės forest between Mančiagirė and Žiūrai villages, Ūla River, rapid, 54.143217° N, 24.441217° E, 22.06.2020, Butkuvienė J. (Ūla 9) BILAS92950	fertile
56	*aquatilis* 3+ (with *circinatus*- and *peltatus*-like copies)	a3 OM692178	D OM721291	C OM721193	Varėna Distr., Trakiškės forest, between Mančiagirė and Žiūrai villages Ūla River, rapid, 54.143217° N, 24.441217° E, 22.06.2020, Butkuvienė J. (Ūla10) BILAS92951	fertile

No.	Taxon	ITS	rpl32-trnL haplotype	petL-psbE haplotype	Locality	Fertility (presence of fruits)
57	*aquatilis* 3+ (with *circinatus*- and *peltatus*-like copies)	a3 OM692179	D OM721292	C OM721194	Varėna Distr., Trakiškės forest, between Mančiagirė and Žiūrai villages, Ūla River, rapid, 54.143217° N, 24.441217° E, 22.06.2020, Butkuvienė J. (Ūla 11) BILAS92952	fertile
58	*fluitans* × *circinatus*	f × c OM692161	A OM721274	A OM721175	Švenčionys Distr., Peršokšna River downstream of Januliškis, rapid, 55.165267° N, 25.839983° E, 26.06.2020, Butkuvienė J. (Peršokšna 2) BILAS92776	sterile
59	*fluitans* × *circinatus*	f × c OM692162	A OM721275	A OM721176	Švenčionys Distr., Peršokšna River downstream of Januliškis, rapid, 55.165267° N, 25.839983° E, 26.06.2020, Butkuvienė J. (Peršokšna 6) BILAS92781	sterile
60	*fluitans* × *circinatus*	f × c OM692163	A OM721276	A OM721177	Švenčionys Distr., Peršokšna River downstream of Januliškis, rapid, 55.165267° N, 25.839983° E, 26.06.2020, Butkuvienė J. (Peršokšna 10) BILAS92785	sterile
61	"*penicillatus*" 2 (*aquatilis* 3+ × (*circinatus* × *fluitans*))	f × c × ? OM692170	D OM721283	C OM721184	Varėna Distr., Merkys River upstream Puvočiai, rapid, 54.13525° N, 24.314183° E, 22.06.2020, Butkuvienė J. (Merkys 9) BILAS92974	sterile
62	"*penicillatus*" 2 (*aquatilis* 3+ × (*circinatus* × *fluitans*))	f × c × ? OM692171	D OM721284	C OM721185	Varėna Distr., Merkys River upstream Puvočiai, rapid, 54.13525° N, 24.314183° E, 22.06.2020, Butkuvienė J. (Merkys 11) BILAS92976	sterile
63	"*penicillatus*" 2 (*aquatilis* 3+ × (*circinatus* × *fluitans*))	f × c × ? OM692172	D OM721285	C OM721186	Varėna Distr., Merkys River upstream Puvočiai, rapid, 54.13525° N, 24.314183° E, 22.06.2020, Butkuvienė J. (Merkys 12) BILAS92977	sterile

No.	Taxon	ITS	rpl32-trnL haplotype	petL-psbE haplotype	Locality	Fertility (presence of fruits)
64	“*penicillatus*” 1 (*aquatilis* 2 ? × (*circinatus* × *fluitans*))	f × c × ? OM692164	A OM721277	A OM721178	Švenčionys Distr., Mera River upstream of Kalviškė village, rapid, 54.9851° N, 25.9087° E, 26.06.2020, Butkuvienė J. (Mera 3) BILAS92750, 92751, 92752, 92753	sterile
65	“*penicillatus*” 1 (*aquatilis* 2 ? × (*circinatus* × *fluitans*))	f × c × ? OM692165	A OM721278	A OM721179	Švenčionys Distr., Mera River upstream of Kalviškė village, rapid, 54.9851° N, 25.9087° E, 26.06.2020, Butkuvienė J. (Mera 4) BILAS92754, 92755	sterile
66	“*penicillatus*” 1 (*aquatilis* 2 ? × (*circinatus* × *fluitans*))	f × c × ? OM692166	A OM721279	A OM721180	Švenčionys Distr., Mera River upstream of Kalviškė village, rapid, 54.9851° N, 25.9087° E, 26.06.2020, Butkuvienė J. (Mera 12) BILAS92762, 92763	sterile
67	*kauffmannii*	k OM692109	C OM721222	C OM721123	Yaroslavl Reg., Nekouz Distr., near Kozlovo village, Sutka River, rapid, 57.904002° N, 38.293316° E, 10.07.2020, Chemeris E. V. (Sutka1 1) IBIW71474	fertile
68	*kauffmannii*	k lc OM692110	C OM721223	C OM721124	Yaroslavl Reg., Nekouz Distr., near Kulotino village, Ild River, riffle, 58.010886° N, 38.169136° E, 14.08.2020, Chemeris E. V. (Ild 1 1) IBIW71477	fertile
69	*kauffmannii*	k lc OM692111	C OM721224	C OM721125	Yaroslavl Reg., Nekouz Distr., near Kulotino village, Ild River, riffle, 58.010886° N, 38.169136° E, 14.08.2020, Chemeris E. V. (Ild 1 2) IBIW71478	fertile
70	*kauffmannii*	k lc OM692112	C OM721225	C OM721126	Yaroslavl Reg., Nekouz Distr., near Andreevskoe village, Ild River, riffle, 58.001453° N, 38.208069° E, 16.08.2020, Chemeris E. V. (Ild 2 1) IBIW71487	fertile

No.	Taxon	ITS	rpl32-trnL haplotype	petL-psbE haplotype	Locality	Fertility (presence of fruits)
71	*kauffmannii*	k<br>OM692113	C<br>OM721226	C<br>OM721127	Yaroslavl Reg., Nekouz Distr., near Andreevskoe village, Ild River, riffle, 58.001453° N, 38.208069° E, 16.08.2020, Chemeris E. V. (Ild 2 2) IBIW71488	fertile
72	*kauffmannii*	k<br>OM692114	C<br>OM721227	C<br>OM721128	Yaroslavl Reg., Myshkin Distr., near Frolovskoe village, Sutka River, riffle, 57.892664° N, 38.284095° E, 19.08.2020, Chemeris E. V. (Sutka 2 1) IBIW71498	fertile
73	*kauffmannii*	k<br>OM692115	C<br>OM721228	C<br>OM721129	Yaroslavl Reg., Myshkin Distr., near Frolovskoe village, Sutka River, riffle, 57.892664° N, 38.284095° E, 19.08.2020, Chemeris E. V. (Sutka 2 4) IBIW71501	fertile
74	*kauffmannii*	k<br>OM692116	C<br>OM721229	C<br>OM721130	Yaroslavl Reg., Myshkin Distr., near Frolovskoe village, Sutka River, riffle, 57.892664° N, 38.284095° E, 19.08.2020, Chemeris E. V. (Sutka 2 7) IBIW71504	fertile
75	*kauffmannii* × *circinatus*	k × c<br>OM692180	C<br>OM721292	C<br>OM721157	Yaroslavl Reg., Uglich Distr., near Falukovo village, Uleima River, riffle, 57.606959° N, 38.470024° E, 23.08.2020, Bobrov A. A., Chemeris E. V. (Uleima 1 1) IBIW71509	sterile
76	*kauffmannii*	k<br>OM692117	C<br>OM721230	C<br>OM721131	Yaroslavl Reg., Uglich Distr., near Falukovo village, Uleima River, riffle, 57.606959° N, 38.470024° E, 23.08.2020, Bobrov A. A., Chemeris E. V. (Uleima 1 2) IBIW71510	fertile
77	*kauffmannii* × *circinatus*	k × c<br>OM692181	C<br>OM721294	C<br>OM721195	Yaroslavl Reg., Uglich Distr., near Falukovo village, Uleima River, riffle, 57.606959° N, 38.470024° E, 23.08.2020, Bobrov A. A., Chemeris E. V. (Uleima 1 10) IBIW71518	sterile

No.	Taxon	ITS	rpl32-trnL haplotype	petL-psbE haplotype	Locality	Fertility (presence of fruits)
78	*kauffmannii* × *circinatus*	k × c OM692182	C OM721295	-	Yaroslavl Reg., Uglich Distr., near Pokrovskoe village, Uleima River, rapid, 57.664873° N, 38.562773° E, 23.08.2020, Bobrov A. A., Chemeris E. V. (Uleima 2 1) IBIW71521	sterile
79	*kauffmannii* × *circinatus*	k × c OM692183	C OM721296	C OM721196	Yaroslavl Reg., Uglich Distr., near Pokrovskoe village, Uleima River, rapid, 57.664873° N, 38.562773° E, 23.08.2020, Bobrov A. A., Chemeris E. V. (Uleima 2 5) IBIW71525	sterile
80	*kauffmannii*	k OM692118	C OM721231	C OM721132	Yaroslavl Reg., Rybinsk Distr., near Iskra Oktyabrya settlement, Korovka River, riffle, 58.014892° N, 38.773403° E, 26.08.2020, Bobrov A. A., Chemeris E. V. (Korovka 4) IBIW71538	fertile
81	*kauffmannii*	k OM692119	C OM721232	C OM721133	Yaroslavl Reg., Rybinsk Distr., near Iskra Oktyabrya settlement, Korovka River, riffle, 58.014892° N, 38.773403° E, 26.08.2020, Bobrov A. A., Chemeris E. V. (Korovka 5) IBIW71539	fertile
82	*kauffmannii*	k OM692120	C OM721233	C OM721134	Yaroslavl Reg., Rybinsk Distr., vicinity of Rybinsk city, Cheremukha River, rapid, 58.008290° N, 38.869577° E, 26.08.2020, Bobrov A. A., Chemeris E. V. (Cheremukha 2) IBIW71547	fertile
83	*kauffmannii*	k OM692121	C OM721234	C OM721135	Yaroslavl Reg., Rybinsk Distr., near Fyodorovskoe village, Koloksha River, riffle, 58.062781° N, 39.143134° E, 29.08.2020, Bobrov A. A., Chemeris E. V. (Koloksha 1) IBIW71557	fertile
84	*kauffmannii*	k OM692122	C OM721235	C OM721136	Yaroslavl Reg., Rybinsk Distr., near Fyodorovskoe village, Koloksha River, riffle, 58.062781° N, 39.143134° E, 29.08.2020, Bobrov A. A., Chemeris E. V. (Koloksha 8) IBIW71564	fertile

No.	Taxon	ITS	rpl32-trnL haplotype	petL-psbE haplotype	Locality	Fertility (presence of fruits)
85	*kauffmannii*	k np OM692123	C OM721236	C OM721137	Ivanovo Reg., Ivanovo Distr., near Pesochnevo village, Chernavka River, riffle, 57.045923° N, 40.848082° E, 09.09.2020, Vinogradova Yu. S., Konotop N. K. (Chernavka 1) IBIW71567	fertile
86	*kauffmannii* × *circinatus*	k × c OM692184	C OM721297	C OM721197	Ivanovo Reg., Ivanovo Distr., near Lomy village, Vostra River, upper part of pond, 56.879389° N, 40.974729° E, 01.10.2020, Vinogradova Yu. S., Konotop N. K. (Vostra 10) IBIW71579	sterile
87	*circinatus*	c OM692096	I OM721210	C OM721111	Yaroslavl Reg., Uglich Distr., Savino village, Uleima River, ford, 57.628140° N, 38.498721° E, 06.06.2013, Belyakov E. A. (Uleima 3) IBIW64404	fertile
88	*circinatus*	c OM692097	I OM721211	C OM721112	Yaroslavl Reg., Uglich Distr., Masalskoe village, Korozhechna River, cattle watering place, reach between former bridges, 57.540746° N, 38.038844° E, 08.09.2018, Movergoz E. A. (Korozhechna 1) IBIW71580	fertile
89	*circinatus*	c OM692098	I OM721212	C OM721113	Ivanovo Reg., Savinskii Distr., near Pelkhovo village, Shizhegda River, reach, 56.547658° N, 41.481324° E, 12.09.2019, Vinogradova Yu. V., Konotop N. K. (Shizhegda 1) IBIW71586	fertile
90	*circinatus*	c OM692099	I OM721213	C OM721114	Ivanovo Reg., Zavolzhsk Distr., Dolmatovskii village, Mera River, small bay, 57.508117° N, 42.327379° E, 29.08.2019, Vinogradova Yu. V., Konotop N. K. (Mera 1) IBIW71588	fertile
91	*kauffmannii*	k OM692124	C OM721237	C OM721138	Yaroslavl Reg., Nekouz Distr., Danilovo village, Ild River, riffle, 57.960715° N, 38.064786° E, 28.05.2013, Movergoz E. A. (Ild 3) IBIW66372	fertile

No.	Taxon	ITS	rpl32-trnL haplotype	petL-psbE haplotype	Locality	Fertility (presence of fruits)
92	*kauffmannii*	k<br>OM692125	C<br>OM721238	C<br>OM721139	Yaroslavl Reg., Myshkin Distr., near Frolovskoe village, Sutka River, 57.892689° N, 38.284132° E, 28.05.2013, Movergoz E. A. (Sutka 2 12) IBIW66387	fertile
93	*kauffmannii*	k<br>OM692126	C<br>OM721239	C<br>OM721140	Ivanovo Reg., Zavolzhsk Distr., near Militino village, Kistega River, riffle, 57.516452° N, 41.979196° E, 28.08.2019, Vinogradova Yu. V., Konotop N. K. (Kistega 1) IBIW71589	fertile
94	*kauffmannii*	k<br>OM692127	C<br>OM721240	C<br>OM721141	Ivanovo Reg., Zavolzhsk Distr., near Vatagi village, Loksha River, 57.458373° N, 41.885448° E, 28.08.2019, Vinogradova Yu. V., Konotop N. K. (Loksha 1) IBIW71590	fertile
95	*kauffmannii* × *circinatus*	k × c<br>OM692185	C<br>OM721298	C<br>OM721191	Yaroslavl Reg., Tutaev Distr., near Vypolzovo village, Urdoma River, downstream bridge, 57.941523° N, 39.495951° E, 23.06.2013, Belyakov E. A. (Urdoma 1) IBIW71591	sterile
96	*kauffmannii* × *circinatus*	k × c<br>OM692186	C<br>OM721299	C<br>OM721198	Yaroslavl Reg., Tutaev Distr., near Vypolzovo village, Urdoma River, downstream bridge, 57.941523° N, 39.495951° E, 23.06.2013, Belyakov E. A. (Urdoma 2) IBIW71592	sterile
97	*kauffmannii* × *circinatus*	k × c<br>OM692187	C<br>OM721300	C<br>OM721199	Yaroslavl Reg., Uglich Distr., Masalskoe village, Korozhechna River, cattle watering place, reach between former bridges, 57.540746° N, 38.038844° E, 08.09.2018, Movergoz E. A. (Korozhechna 2) IBIW71581	sterile
98	*kauffmannii* × *circinatus*	k × c<br>OM692188	—	C<br>OM721200	Yaroslavl Reg., Uglich Distr., Masalskoe village, Korozhechna River, cattle watering place, reach between former bridges, 57.540746° N, 38.038844° E, 08.09.2018, Movergoz E. A. (Korozhechna 3) IBIW71582	sterile

No.	Taxon	ITS	rpl32-trnL haplotype	petL-psbE haplotype	Locality	Fertility (presence of fruits)
99	*kauffmannii* × *circinatus*	k × c OM692189	C OM721301	C OM721201	Yaroslavl Reg., Uglich Distr., Masalskoe village, Korozhechna River, cattle watering place, reach between former bridges, 57.540746° N, 38.038844° E, 08.09.2018, Movergoz E. A. (Korozhechna 4) IBIW71583	sterile
100	*kauffmannii* × *circinatus*	k × c OM692190	—	C OM721202	Yaroslavl Reg., Uglich Distr., Masalskoe village, Korozhechna River, cattle watering place, reach between former bridges, 57.540746° N, 38.038844° E, 08.09.2018, Movergoz E. A. (Korozhechna 5) IBIW71584	sterile
101	*kauffmannii* × *circinatus*	k × c OM692191	C OM721302	C OM721203	Yaroslavl Reg., Uglich Distr., Masalskoe village, Korozhechna River, cattle watering place, reach between former bridges, 57.540746° N, 38.038844° E, 08.09.2018, Movergoz E. A. (Korozhechna 6) IBIW71585	sterile
102	*kauffmannii* × *circinatus*	k × c OM692192	C OM721303	C OM721204	Ivanovo Reg., Savinskii Distr., near Pelkhovo village, Shizhegda River, reach, 56.547658° N, 41.481324° E, 12.09.2019, Vinogradova Yu. V., Konotop N. K. (Shizhegda 2) IBIW71587	sterile

Table S2. Sequence variation in the nuclear ITS region from *Ranunculus aquatilis* s.l., *R. baudotii*, *R. circinatus*, *R. fluitans*, *R. kauffmannii*, *R. peltatus*, *R. penicillatus*, *R. schmalhausenii*, *R. trichophyllus* s.l. and hybrids with DNA sample reference numbers (GenBank accessions or according Table S1). Polymorphic nucleotide sites (SNPs) are coded using the IUPAC code. Polymorphisms characteristic for within-species forms are gray shed underlined, polymorphisms characteristic for *R. circinatus* × *R. fluitans* are light green shed underlined, for *R. circinatus* × *R. fluitans*, *R. circinatus* × *R. kauffmannii*, *R. aquatilis* 3, *R. aquatilis* 3 × *R. circinatus* and two derivative *R.* "*penicillatus*" forms are darker green shed underlined, for *R. circinatus* × *R. kauffmannii*, *R. aquatilis* 3 and *R. aquatilis* 3 × *R. circinatus* and derivative *R.* "*penicillatus*" 1 form are light blue shed underlined, for *R. aquatilis* 2, *R. aquatilis* 3 × *R. circinatus* and two derivative *R.* "*penicillatus*" forms are yellow shed underlined, and for *R. aquatilis* 2, 3, *R. aquatilis* 3 × *R. circinatus* and derivative *R.* "*penicillatus*" 1 are light brown shed underlined. Hybrid names were given regarding the crossing direction: maternal species × paternal species.

Taxon	Sample or GB accession no.	5	13	30	32	35	43	46	48	49	55	60	63	64	67	72	76	78	85	86	89	97	98	112	124	169	180	191	192	201	202	399	401-405	418	419	449	450	465	481	501	512	529	559
baudotii (northern lineage)	KF719058 Estonia, AY680068 France	C	C	G	G	C	G	C	T	A	C	T	T	C	C	C	T	A	T	G	C	T	T	C	G	C	C	C	G	A	C	C	- - - - -	G	C	C	T	C	G	C	T	G	C
fluitans	KR996526 Poland, 25, 27	C	C	G	G	C	A	C	C	A	C	T	T	C	C	C	A	A	T	G	C	T	T	C	G	C	C	C	A	A	C	C	- - - - -	G	C	C	T	C	G	C	T	G	T
circinatus 1	KF719061 Poland, 90	C	T	–	G	T	G	T	C	G	C	C	T	C	C	C	T	G	C	A	C	A	C	C	G	C	C	T	A	G	C	C	- - - - -	A	T	T	T	C	G	C	A	A	C
circinatus 2	MZ338333 C Russia	C	T	–	G	T	G	T	C	G	C	C	T	C	C	C	T	G	C	G	T	A	C	C	G	C	T	T	A	G	C	C	- - - - -	A	T	T	T	C	G	C	A	A	C
circinatus 3	87, 88	C	T	–	G	T	G	T	C	G	C	C	T	C	C	C	T	G	C	G	C	A	C	C	G	C	C	T	A	G	C	C	- - - - -	A	T	T	T	C	G	C	A	A	C
circinatus 3+	89	C	T	–	G	T	G	T	C	G	C	C	T	C	C	C	T	G	C	**R**	C	A	C	C	G	C	C	T	A	G	C	C	- - - - -	A	T	T	T	C	G	C	A	A	C
circinatus 3++	37, 38 (39 no ITS)	C	T	–	G	T	G	T	C	G	C	C	T	C	C	C	T	G	C	**R**	C	A	C	C	G	C	C	T	A	G	**Y**	C	- - - - -	A	T	T	T	C	G	C	A	A	C

Taxon	Sample or GB accession no.	5	13	30	32	35	43	46	48	49	55	60	63	64	67	72	76	78	85	86	89	97	98	112	124	169	180	191	192	201	202	399	401-405	418	419	449	450	465	481	501	512	529	559
aquatilis 1 (cf. *trichophyllus* B)	KF719055 Poland, KF719056 Croatia,	C	T	–	G	T	G	T	C	G	C	C	T	T	T	C	T	G	T	G	C	T	T	C	G	C	C	C	A	G	T	C	- - - - -	G	C	C	T	T	G	T	T	A	C
aquatilis 1+ (cf. *trichophyllus* B)	40—42	C	T	–	G	T	G	T	C	G	C	C	T	T	T	C	T	G	T	G	C	T	T	C	G	C	C	C	A	G	T	C	- - - - -	G	C	C	T	T	G	C	T	A	C
trichophyllus B	MW430946 Czech Rep.	C	T	–	G	T	G	T	C	G	C	C	T	T	T	C	T	G	T	G	C	T	T	C	G	C	C	C	A	G	T	C	- - - - -	G	C	C	T	T	G	C	T	A	C
peltatus	KF719068 Poland	C	T	–	G	T	G	T	C	A	C	C	T	C	C	T	T	G	C	G	C	T	T	C	G	C	C	C	A	G	T	C	- - - - -	G	C	C	C	C	A	C	T	A	C
schmalhausenii	KR996541 NW Russia	C	T	–	A	T	G	T	C	A	A	C	T	C	C	C	T	G	C	G	C	T	T	C	G	C	C	C	A	G	T	C	- - - - -	G	C	C	C	C	**R**	C	T	A	C
schmalhausenii	KR996559 NW Russia	C	T	–	A	T	G	T	C	A	**W**	C	T	C	C	C	T	G	C	G	C	T	T	C	G	C	C	C	A	G	T	C	- - - - -	G	C	C	C	C	**R**	C	T	A	C
penicillatus	KR996528 Poland	C	T	–	**R**	T	G	T	C	A	**Y**	C	T	C	C	**Y**	T	G	C	G	C	T	T	C	G	C	C	C	A	G	T	C	- - - - -	G	C	C	C	C	A	C	T	A	C
trichophyllus 1	KF719073 Poland	T	T	–	G	T	G	T	C	A	C	C	C	C	C	C	T	G	C	G	C	T	T	C	A	C	C	C	A	G	T	C	- - - - -	G	C	C	C	C	A	C	T	A	C
trichophyllus 2	KR996565 Poland	C	T	–	A	T	G	T	C	A	C	C	T	C	C	C	T	G	C	G	C	T	T	T	G	T	C	C	A	G	T	C	- - - - -	G	C	C	C	C	A	C	T	A	C

Taxon	Sample or GB accession no.	5	13	30	32	35	43	46	48	49	55	60	63	64	67	72	76	78	85	86	89	97	98	112	124	169	180	191	192	201	202	399	401-405	418	419	449	450	465	481	501	512	529	559
trichophyllus 4	KR996568 Kamchatka, MZ338327 NE Yakutia	C	T	–	G	T	G	T	C	A	C	C	T	C	C	C	T	G	C	G	C	T	T	C	G	C	C	C	A	G	T	T	- - - - -	G	C	C	C	C	A	C	T	A	C
kauffmannii	MZ338329 C Russia, 1—3, 94	C	T	–	**R**	T	G	T	C	A	C	C	**Y**	C	C	C	T	G	C	G	C	T	T	**Y**	**R**	**Y**	C	C	A	G	T	C	- - - - -	G	C	C	C	C	A	C	T	A	C
kauffmannii	67, 74	T	T	–	**R**	T	G	T	C	A	C	C	**Y**	C	C	C	T	G	C	G	C	T	T	**Y**	**R**	**Y**	C	C	A	G	T	C	- - - - -	G	C	C	C	C	A	C	T	A	C
kauffmannii	68, 70	T	T	–	**R**	T	G	T	C	A	C	C	**Y**	C	C	C	T	G	C	G	C	T	T	**Y**	**R**	**Y**	C	C	A	G	T	C	CCCCA	G	C	C	C	C	A	C	T	A	C
kauffmannii	69	**Y**	T	–	**R**	T	G	T	C	A	C	C	**Y**	C	C	C	T	G	C	G	C	T	T	**Y**	**R**	**Y**	C	C	A	G	T	C	CCCCA	G	C	C	C	C	A	C	T	A	C
kauffmannii	71, 72, 73	**Y**	T	–	**R**	T	G	T	C	A	C	C	**Y**	C	C	C	T	G	C	G	C	T	T	**Y**	**R**	**Y**	C	C	A	G	T	C	- - - - -	G	C	C	C	C	A	C	T	A	C
kauffmannii	10	T	T	–	**R**	T	G	T	C	A	C	C	**Y**	C	C	C	T	G	C	G	C	T	T	C	**R**	**Y**	C	C	A	G	T	C	- - - - -	G	C	C	C	C	A	C	T	A	C
kauffmannii	12	T	T	–	G	T	G	T	C	A	C	C	**Y**	C	C	C	T	G	C	G	C	T	T	C	**R**	**Y**	C	C	A	G	T	C	- - - - -	G	C	C	C	C	A	C	T	A	C
kauffmannii	80, 81, 82, 83	**Y**	T	–	**R**	T	G	T	C	A	C	C	**Y**	C	C	C	T	G	C	G	C	T	T	C	**R**	**Y**	C	C	A	G	T	C	- - - - -	G	C	C	C	C	A	C	T	A	C
kauffmannii	84	C	T	–	**R**	T	G	T	C	A	C	C	**Y**	C	C	C	T	G	C	G	C	T	T	C	**R**	**Y**	C	C	A	G	T	C	- - - - -	G	C	C	C	C	A	C	T	A	C
kauffmannii	11	T	T	–	**R**	T	G	T	C	A	C	C	**Y**	C	C	C	T	G	C	G	C	T	T	C	**R**	C	C	C	A	G	T	C	- - - - -	G	C	C	C	C	A	C	T	A	C
kauffmannii	76	C	T	–	**R**	T	G	T	C	A	C	C	**Y**	C	C	C	T	G	C	G	C	T	T	C	A	**Y**	C	C	A	G	T	C	- - - - -	G	C	C	C	C	A	C	T	A	C
kauffmannii	91	C	T	–	A	T	G	T	C	A	C	C	T	C	C	C	T	G	C	G	C	T	T	**Y**	G	T	C	C	A	G	T	C	- - - - -	G	C	C	C	C	A	C	T	A	C

Taxon	Sample or GB accession no.	5	13	30	32	35	43	46	48	49	55	60	63	64	67	72	76	78	85	86	89	97	98	112	124	169	180	191	192	201	202	399	401-405	418	419	449	450	465	481	501	512	529	559
kauffmannii	92	T	T	–	G	T	G	T	C	A	C	C	**Y**	C	C	C	T	G	C	G	C	T	T	C	A	C	C	C	A	G	T	C	- - - - -	G	C	C	C	C	A	C	T	A	C
kauffmannii	85	C	T	–	G	T	G	T	C	A	C	C	C	C	C	C	T	G	C	G	C	T	T	C	A	C	C	C	A	G	T	C	- - - - -	G	C	C	C	C	A	C	T	A	C
kauffmannii	93	C	T	–	A	T	G	T	C	A	C	C	T	C	C	C	T	G	C	G	C	T	T	C	G	T	C	C	A	G	T	C	- - - - -	G	C	C	C	C	A	C	T	A	C
fluitans × *circinatus*	7—9, 13—15, 22—23, 26, 28—30, 34—36, 43, 45, 47, 49, 50, 58—60	C	**Y**	**G/–**	G	**Y**	**R**	**Y**	C	**R**	C	**Y**	T	C	C	C	**W**	**R**	**Y**	G	C	**W**	**Y**	C	G	C	C	**Y**	A	**R**	**Y**	C	- - - - -	**R**	**Y**	**Y**	T	C	G	C	**W**	**R**	**Y**
fluitans × *circinatus*	16—21, 31—33, 44, 46, 48	C	**Y**	**G/–**	G	**Y**	**R**	**Y**	C	**R**	C	**Y**	T	C	C	C	**W**	**R**	**Y**	G	C	**W**	**Y**	C	G	C	C	**Y**	A	**R**	C	C	- - - - -	**R**	**Y**	**Y**	T	C	G	C	**W**	**R**	**Y**
"*penicillatus*" 1 (*aquatilis* 2 ? × (*circinatus* × *fluitans*))	64—66	C	**Y**	**G/–**	G	**Y**	**R**	**Y**	C	**R**	C	**Y**	T	C	C	**Y**	**W**	**R**	**Y**	G	C	**W**	**Y**	C	G	C	C	**Y**	A	**R**	**Y**	C	- - - - -	**R**	**Y**	C	**Y**	**Y**	**R**	C	**W**	**R**	**Y**
"*penicillatus*" 2 (*aquatilis* 3+ × (*circinatus* × *fluitans*))	51—53, 61—63	C	**Y**	**G/–**	G	**Y**	**R**	**Y**	C	**R**	C	**Y**	T	**Y**	**Y**	C	**W**	**R**	**Y**	G	C	**W**	**Y**	C	G	C	C	**Y**	A	**R**	**Y**	C	- - - - -	**R**	**Y**	**Y**	T	**Y**	G	C	**W**	**R**	**Y**
aquatilis 2 (with *peltatus*-like copy)	MW430819 Czech Rep.	C	T	–	G	T	G	T	C	**R**	C	C	T	**Y**	**Y**	**Y**	T	G	**Y**	G	C	T	T	C	G	C	C	C	A	G	T	C	- - - - -	G	C	C	**Y**	**Y**	**R**	**Y**	T	A	C

Taxon	Sample or GB accession no.	5	13	30	32	35	43	46	48	49	55	60	63	64	67	72	76	78	85	86	89	97	98	112	124	169	180	191	192	201	202	399	401-405	418	419	449	450	465	481	501	512	529	559
aquatilis 3 (with *circinatus*-like copy)	MW430811 Czech Rep.	C	T	–	G	T	G	T	C	**R**	C	C	T	C	C	C	T	G	C	G	C	**W**	**Y**	C	G	C	C	**Y**	A	**R**	**Y**	C	- - - - -	**R**	**Y**	C	T	C	**R**	C	**W**	A	C
aquatilis 3+ (with *circinatus*- and *peltatus*-like copies)	54	C	T	–	G	T	G	T	C	**R**	C	C	T	C	C	**Y**	T	G	C	G	C	**W**	**Y**	C	G	C	C	**Y**	A	**R**	**Y**	C	- - - - -	**R**	**Y**	C	**Y**	C	**R**	C	**W**	A	C
aquatilis 3+ (with *circinatus*- and *peltatus*-like copies)	56	C	T	–	G	T	G	T	C	**R**	C	C	T	**Y**	C	**Y**	T	G	C	G	C	**W**	**Y**	C	G	C	C	**Y**	A	**R**	**Y**	C	- - - - -	**R**	**Y**	C	**Y**	C	**R**	C	**W**	A	C
aquatilis 3+ (with *circinatus*- and *peltatus*-like copies)	55, 57	C	T	–	G	T	G	T	C	**R**	C	C	T	**Y**	C	**Y**	T	G	**Y**	G	C	**W**	**Y**	C	G	C	C	**Y**	A	**R**	**Y**	C	- - - - -	**R**	**Y**	C	**Y**	C	**R**	C	**W**	A	C
aquatilis 3+ × *circinatus*	4	C	T	–	**R**	T	G	T	C	**R**	C	C	T	C	C	**Y**	T	G	C	G	C	**W**	**Y**	C	G	**Y**	C	**Y**	A	**R**	**Y**	C	- - - - -	**R**	**Y**	C	**Y**	C	**R**	C	**W**	A	C
aquatilis 3+ × *circinatus*	5	C	T	–	**R**	T	G	T	C	**R**	C	C	T	C	C	**Y**	T	G	C	G	C	**W**	**Y**	C	G	**Y**	C	**Y**	A	**R**	**Y**	C	- - - - -	**R**	**Y**	C	**Y**	**Y**	**R**	C	**W**	A	C
aquatilis 3+ × *circinatus*	6	C	T	–	**R**	T	G	T	C	**R**	C	C	T	**Y**	C	**Y**	T	G	C	G	C	**W**	**Y**	C	G	**Y**	C	**Y**	A	**R**	**Y**	C	- - - - -	**R**	**Y**	C	**Y**	**Y**	**R**	C	**W**	A	C
kauffmannii × *circinatus*	75, 77—79	C	T	–	G	T	G	T	C	**R**	C	C	**Y**	C	C	C	T	G	C	**R**	C	**W**	**Y**	C	**R**	C	C	**Y**	A	G	**Y**	C	- - - - -	**R**	**Y**	**Y**	**Y**	C	**R**	C	**W**	A	C

Taxon	Sample or GB accession no.	5	13	30	32	35	43	46	48	49	55	60	63	64	67	72	76	78	85	86	89	97	98	112	124	169	180	191	192	201	202	399	401-405	418	419	449	450	465	481	501	512	529	559
kauffmannii × *circinatus*	86	**Y**	T	–	**R**	T	G	T	C	**R**	C	C	T	C	C	C	T	G	C	**R**	C	**W**	**Y**	**Y**	**R**	**Y**	C	**Y**	A	G	**Y**	C	- - - - -	**R**	**Y**	**Y**	**Y**	C	**R**	C	**W**	A	C
kauffmannii × *circinatus*	95, 96, 102	C	T	–	G	T	G	T	C	**R**	C	C	**Y**	C	C	C	T	G	C	G	C	**W**	**Y**	C	**R**	C	C	**Y**	A	G	**Y**	C	- - - - -	**R**	**Y**	**Y**	**Y**	C	**R**	C	**W**	A	C
kauffmannii × *circinatus*	97—101	**Y**	T	–	G	T	G	T	C	**R**	C	C	**Y**	C	C	C	T	G	C	**R**	C	**W**	**Y**	C	**R**	C	C	**Y**	A	G	**Y**	C	- - - - -	**R**	**Y**	**Y**	**Y**	C	**R**	C	**W**	A	C

Table S3. Sequence variation in the plastid rpl32-trnL region from the studied *Ranunculus* (*Batrachium*) taxa.
Details on haplotypes of the sequences see in Table S1 caption. Hybrid names were given regarding the crossing direction: maternal species × paternal species.

Haplotype	Taxon	DNA sample no.	134	229	282—285	391	406	434	455	462	488	500	525	546	559	570	578	638	650	661	703	715	725	726	765	775	785	841
A	*R. aquatilis* 1<br>*R. fluitans*<br>*R. fluitans* × *R. circinatus*<br><br>*R.* "*penicillatus*" 1 (*R. aquatilis* 2 ? × (*R. circinatus* × *R. fluitans*))	40—42<br>25, 27<br>7—9, 13—24, 26, 28—36, 43—50, 58—60<br>64—66	C	C	ATAT	G	C	A	A	G	G	C	G	A	G	C	C	G	A	C	C	G	G	G	G	G	G	C
C	*R. kauffmannii*<br><br>*R. kauffmannii* × *R. circinatus*	1—3, 67—74, 76, 80—85, 91—93<br>75, 77—79, 86, 95—97, 99, 101, 102	C	C	ATAT	G	C	A	A	G	G	C	G	T	A	A	C	G	A	C	G	G	G	G	G	G	G	C
D	*R. kauffmannii*<br>*R.* "*penicillatus*" 2 (*R. aquatilis* 3 × (*R. circinatus* × *R. fluitans*))<br>*R. aquatilis* 3	10, 11<br>51—53, 61—63<br><br>54—57	C	C	ATAT	G	C	C	A	G	G	C	G	A	A	A	C	G	A	C	G	G	G	G	G	G	G	C
D+	*R. aquatilis* 3 × *R. circinatus*	4—6	C	C	ATAT	A	C	C	A	G	G	C	G	A	A	A	C	G	A	C	G	G	G	G	G	G	G	C
I	*R. circinatus*	37—39, 87—90	A	T	- - - -	G	T	A	T	T	A	G	T	A	G	A	T	T	C	A	C	A	A	T	A	A	A	T

Table S4. Sequence variation in the plastid petL-psbE region from the studied *Ranunculus* (*Batrachium*) taxa.
Details on haplotypes of the sequences see in Table S1 caption. Hybrid names were given regarding the crossing direction: maternal species × paternal species.

Haplotype	Taxon	DNA sample no.	349	421	717	814	817—821	903	951
A	*R. aquatilis* 1	41, 42	C	-	C	T	- - - - -	G	C
	R. fluitans	25, 27							
	R. fluitans × *R. circinatus*	8, 9, 13—24, 26, 28—36, 43—50, 58—60							
	R. “*penicillatus*” 1 (*R. aquatilis* 2 ? × (*R. circinatus* × *R. fluitans*))	64—66							
C	*R. kauffmannii*	1—3, 10—12, 67—74, 76, 80—85, 91—94	C	T	C	C	- - - - -	G	C
	R. kauffmannii × *R. circinatus*	75, 77, 79, 86, 95—102							
	R. “*penicillatus*” 2 (*R. aquatilis* 3 × (*R. circinatus* × *R. fluitans*))	51—53, 61—63							
	R. aquatilis 3	54—57							
	R. aquatilis 3 × *R. circinatus*	4—6							
F	*R. circinatus*	37—39, 87—90	A	T	A	C	AACCC	A	T

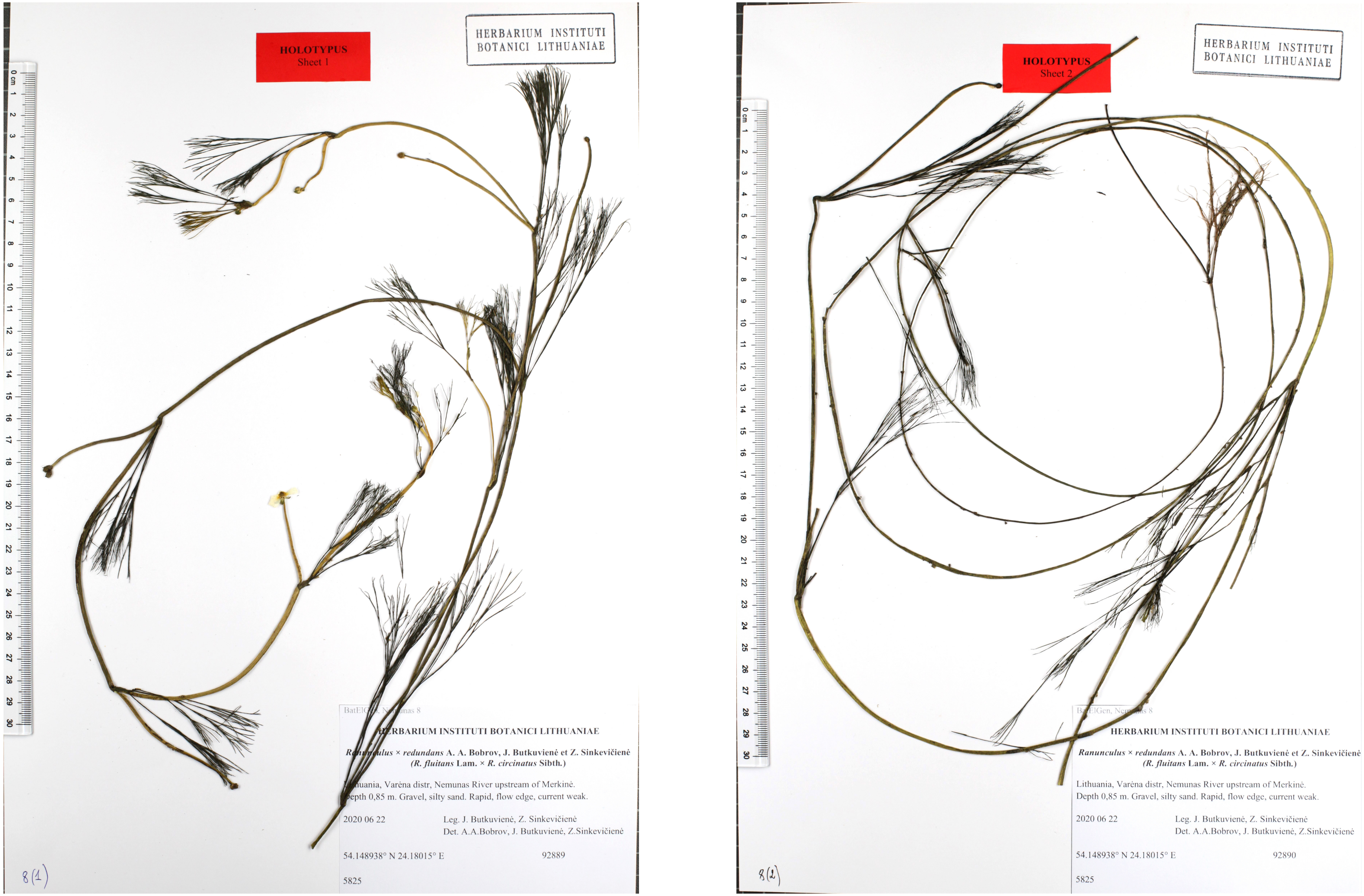

Fig. S1. Holotype of *Ranunculus* × *redundans* (*R. circinatus* × *R. fluitans*) preserved in BILAS.

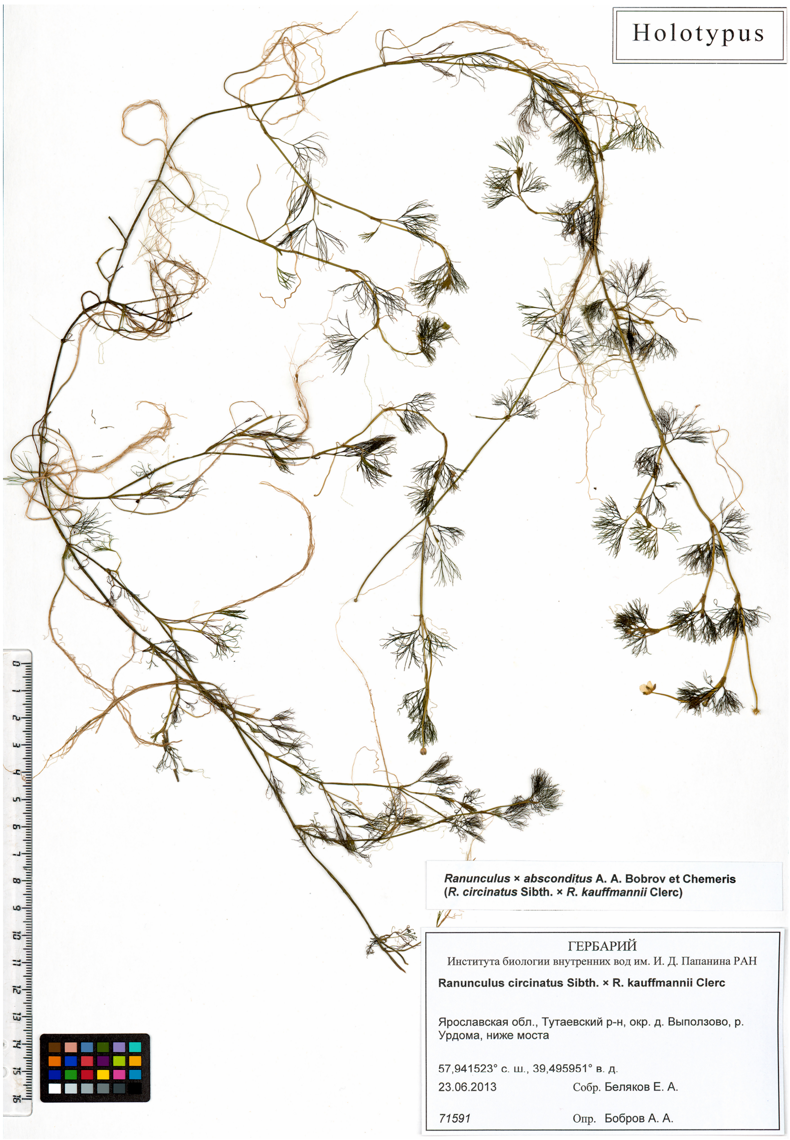

Fig. S2. Holotype of *Ranunculus* × *absconditus* (*R. circinatus* × *R. kauffmannii*) preserved in IBIW.
